# Deletion of *Abi3* gene locus exacerbates neuropathological features of Alzheimer’s disease in a mouse model of Aβ amyloidosis

**DOI:** 10.1126/sciadv.abe3954

**Published:** 2021-11-03

**Authors:** Hande Karahan, Daniel C. Smith, Byungwook Kim, Luke C. Dabin, Md Mamun Al-Amin, H. R. Sagara Wijeratne, Taylor Pennington, Gonzalo Viana di Prisco, Brianne McCord, Peter Bor-chian Lin, Yuxin Li, Junmin Peng, Adrian L. Oblak, Shaoyou Chu, Brady K. Atwood, Jungsu Kim

**Affiliations:** 1 Stark Neurosciences Research Institute, Indiana University School of Medicine, Indianapolis, IN 46202, USA.; 2 Department of Medical and Molecular Genetics, Indiana University School of Medicine, Indianapolis, IN 46202, USA.; 3 Medical Neuroscience Graduate Program, Indiana University School of Medicine, Indianapolis, IN 46202, USA.; 4 Department of Biochemistry and Molecular Biology, Indiana University School of Medicine, Indianapolis, IN 46202, USA.; 5 Department of Pharmacology and Toxicology, Indiana University School of Medicine, Indianapolis, IN 46202, USA.; 6 Departments of Structural Biology and Developmental Neurobiology, St. Jude Children’s Research Hospital, Memphis, TN 38105, USA.; 7 Center for Proteomics and Metabolomics, St. Jude Children’s Research Hospital, Memphis, TN 38105, USA.; 8 Department of Radiology and Imaging Sciences, Indiana University School of Medicine, Indianapolis, IN 46202, USA.; 9 Division of Clinical Pharmacology, Indiana University School of Medicine, Indianapolis, IN 46202, USA.

## Abstract

Recently, large-scale human genetics studies identified a rare coding variant in the *ABI3* gene that is associated with an increased risk of Alzheimer’s disease (AD). However, pathways by which ABI3 contributes to the pathogenesis of AD are unknown. To address this question, we determined whether loss of ABI3 function affects pathological features of AD in the 5XFAD mouse model. We demonstrate that the deletion of *Abi3* locus significantly increases amyloid β (Aβ) accumulation and decreases microglia clustering around the plaques. Furthermore, long-term potentiation is impaired in *5XFAD;Abi3* knockout (“*Abi3^−/−^*”) mice. Moreover, we identified marked changes in the proportion of microglia subpopulations in *Abi3^−/−^* mice using a single-cell RNA sequencing approach. Mechanistic studies demonstrate that *Abi3* knockdown in microglia impairs migration and phagocytosis. Together, our study provides the first in vivo functional evidence that loss of ABI3 function may increase the risk of developing AD by affecting Aβ accumulation and neuroinflammation.

## Teaser

A cytoskeleton-associated protein regulates microglial function and affects Alzheimer’s disease phenotypes.

## INTRODUCTION

Alzheimer’s disease (AD) is the most common cause of dementia. It is pathologically characterized by abnormal accumulation of amyloid β (Aβ) as amyloid plaques, hyperphosphorylated tau in the form of neurofibrillary tangles, and neuroinflammation in the brain (*1*). Mounting evidence suggests that neuroinflammation in AD is not simply a response to neurodegeneration but plays a crucial role in disease pathogenesis (*2*, *3*). Genome-wide association studies (GWASs) of AD have identified many risk genes that are highly or selectively expressed in microglia, the predominant immune cells of the brain (*4*). A number of studies have supported the importance of microglia in homeostatic and pathological conditions, including AD (*5*, *6*). Specifically, microglia can promote clearance of Aβ through phagocytosis and regulate brain immunity by secreting inflammatory cytokines in AD (*7*). Because there have been numerous studies demonstrating both protective and detrimental effects of microglia in AD, how microglial AD risk genes regulate the function of these cells remains elusive. To this end, determining the functional consequences of these microglia genetic risk factors in AD can provide a valuable insight into the pathobiology of the disease. Moreover, these findings can lead to identification of new drug targets for AD.

Recently, a large-scale human genetics study identified a rare coding variant in Abelson interactor family member 3 (*ABI3*) gene (rs616338, S209P, *P* = 4.56 × 10^−10^, odss ratio = 1.43), and this variant was associated with increased risk of late-onset AD (LOAD) (*8*). Subsequently, this association was replicated by independent groups (*9*, *10*). However, whether this variant in the *ABI3* gene causes gain- or loss-of-function has not been known yet. Similar to newly identified other AD risk variants (e.g., *TREM2*, *SPI1*, and *MS4A6A*), *ABI3* is highly expressed in microglia (*8*, *9*, *11*). ABI3 has been identified as a component of WASP-family verprolin homologous protein (WAVE) regulatory complex in several cancer cells (*12*, *13*). This complex regulates actin cytoskeleton organization and participates in cytokinesis, migration, endocytosis, and phagocytosis (*14*, *15*). Although the molecular and functional characteristics of ABI3 have been studied in a few cancer cell culture models (*16*, *17*), its physiological role in the brain, especially in microglia, and the mechanisms by which ABI3 contributes to the etiology of AD are unknown.

To fill this critical knowledge gap, we investigated the role of *Abi3* in the pathological features of AD, such as Aβ accumulation, gliosis, and synaptic dysfunctions, using the 5XFAD mouse model. We found that deletion of *Abi3* locus significantly increased Aβ levels and amyloid plaques and decreased microglia clustering around the plaques. Transcriptomic and gene enrichment analyses identified immune response as the top biological pathway that is dysregulated by deletion of the *Abi3* gene locus. Using a single-cell RNA sequencing (scRNA-seq) approach, we found a marked shift in the proportion of certain microglia subpopulations in *Abi3* knockout (*Abi3^−/−^*) mice. In addition, we demonstrated that cytokine levels were altered and synaptic dysfunction was exacerbated in the brains of *Abi3^−/−^* mice. Furthermore, in vitro mechanistic analyses revealed that knockdown of *Abi3* gene impairs migration and phagocytosis of microglia. Our data suggest that *Abi3* may affect the pathogenesis of AD by regulating microglia function and Aβ pathology.

## RESULTS

### Expression pattern of ABI3 in human LOAD brains

The level of *ABI3* mRNA was assessed in human brain cortices from patients with LOAD and control groups (*8*). *ABI3* expression was enriched in microglia and was higher in LOAD human cortex compared to controls. However, after correcting for the difference in cell-type composition between brain samples, the increase in the *ABI3* mRNA level was not statistically significant, indicating microgliosis-dependent increase in *ABI3* level in AD brain (*8*). It is still unknown whether these transcriptomic changes are reflected in the protein levels. Therefore, we analyzed the level of ABI3 protein in the human proteomics dataset from parahippocampal cortices of patients with AD in the Mount Sinai Brain Bank study (table S1) (*18*). The level of ABI3 protein was significantly increased in brains of patients with AD compared to cognitively normal samples (fig. S1A). However, similar to transcriptomic analysis, there was no statistically significant difference between the groups after normalizing the data based on the difference in the proportion of different cell types in the brains (fig. S1B). Because *ABI3* is highly expressed in microglia, it is likely that the increase in the ABI3 protein level before normalization was due to the increased number of microglia, one of the characteristic features of AD. Based on these expression association studies, it is not possible to predict whether an increase in gene expression is a detrimental or compensatory beneficial mechanism. For example, another microglial AD risk gene, *TREM2*, was found to be up-regulated in brains of patients with AD (*19*). Functional studies demonstrated that deletion of *Trem2* altered amyloid pathology in opposite directions depending on the stage of the disease (*20*–*23*), but overexpression of *TREM2* reduced the pathology in transgenic mouse models of amyloidosis (*24*).

### Soluble and insoluble Aβ levels are increased in *Abi3^−/−^* mice

**Fig. 1.**
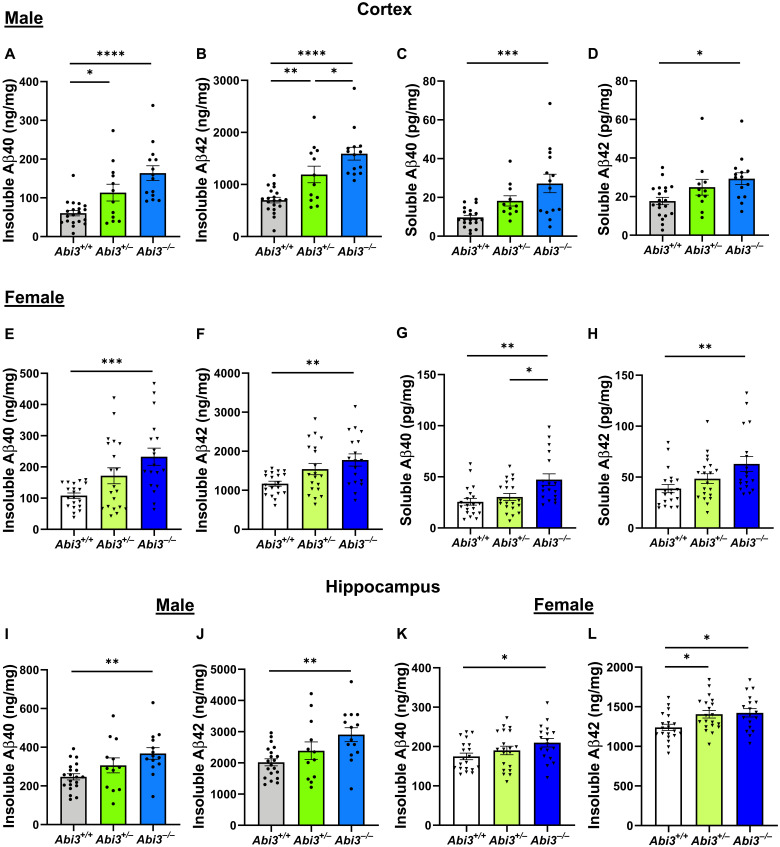
Aβ accumulation increases in the cortex and hippocampus of male and female *Abi3^−/−^* mice. Insoluble Aβ40 and Aβ42 levels were measured in the guanidine fraction (**A** and **B**) and soluble Aβ40 and Aβ42 levels were measured in the phosphate-buffered saline (PBS) fraction (**C** and **D**) of 5XFAD mouse cortices using Meso Scale Discovery (MSD) electrochemiluminescence assay. Insoluble Aβ40 (A) and Aβ42 (B) levels were increased in the cortices of 8-month-old male *Abi3^+/−^* and *Abi3^−/−^* mice compared to *Abi3^+/+^* mice. Soluble Aβ40 (C) and Aβ42 (D) levels were increased in the cortices of 8-month-old male *Abi3^−/−^* mice compared to *Abi3^+/+^* mice. Insoluble Aβ40 (**E**) and Aβ42 (**F**) levels were increased in the cortices of 8-month-old female *Abi3^−/−^* mice compared to *Abi3^+/+^* mice. Soluble Aβ40 (**G**) and Aβ42 (**H**) levels were increased in the cortices of 8-month-old female *Abi3^−/−^* mice compared to *Abi3^+/+^* mice. (**I** to **L**) Insoluble Aβ40 and Aβ42 levels were measured in the hippocampus. Male *Abi3^−/−^* mice accumulated higher levels of (I) Aβ40 and (J) Aβ42. Similarly, female *Abi3^−/−^* mice had higher levels of (K) Aβ40 and (L) Aβ42 (*Abi3^+/+^*, *n* = 20 male and 20 female; *Abi3^+/−^*, *n* = 12 male and 20 female; *Abi3^−/−^*, *n* = 14 male and 18 female). Data represent means ± SEM. One-way analysis of variance (ANOVA) and Tukey’s multiple comparisons test; *P < 0.05, **P < 0.01, ***P < 0.001, and ****P < 0.0001. See also fig. S3.

To investigate the role of *Abi3* in pathological features of AD, we bred *Abi3* knockout mice with 5XFAD mice, a transgenic mouse model of Aβ amyloidosis. We generated 5XFAD mice expressing two copies of *Abi3* (*5XFAD;Abi3^+/+^*, referred to as *Abi3^+/+^*), one copy of *Abi3* (*5XFAD;Abi3^+/−^*, referred to as *Abi3^+/−^*), and no expression of *Abi3* (*5XFAD;Abi3^−/−^*, referred to as *Abi3^−/−^*). Using an SD of 400, an effect size of 40% for insoluble Aβ42 levels, a power of 0.8, and *P* < 0.05, we aimed to generate at least 12 mice per sex for each genotype. Because there is no good commercially available ABI3 antibody that can detect endogenously expressed ABI3 protein, we confirmed the deletion of *Abi3* gene in the brains of these mice by quantitative polymerase chain reaction (qPCR) (fig. S2, A and B). To determine the effect of *Abi3* gene dosage on Aβ accumulation, we measured the levels of soluble and insoluble Aβ40 and Aβ42 in the brains of 8-month-old *Abi3^+/+^*, *Abi3^+/−^*, and *Abi3^−/−^* mice using Aβ electrochemiluminescence assay. Because 5XFAD mice are known to have a sex-dependent difference in the extent of amyloid accumulation (*25*), we designed our study to consider sex difference, analyzing the results separately between male and female mice. Loss of *Abi3* expression markedly increased the levels of insoluble, guanidine-extracted, Aβ40 and Aβ42 in the cortical regions of 8-month-old male 5XFAD mice in a gene dose–dependent manner (Fig. 1, A and B). Insoluble Aβ40 levels were increased by 1.9- and 2.7-fold in male *Abi3^+/−^* and *Abi3^−/−^* mouse cortices compared to *Abi3^+/+^* mice, respectively (Fig. 1A). Similarly, Aβ42 levels were increased by 1.7- and 2.3-fold in *Abi3^+/−^* and *Abi3^−/−^* mice compared to *Abi3^+/+^* mice, respectively (Fig. 1B). In addition to insoluble Aβ levels, soluble Aβ40 levels were increased by 1.4- and 2.1-fold and soluble Aβ42 levels were increased by 1.4- and 1.6-fold in male *Abi3^+/−^* and *Abi3^−/−^* mouse cortices compared to *Abi3^+/+^* mice, respectively (Fig. 1, C and D). In the female groups, the levels of insoluble Aβ40 and Aβ42 were also increased in the cortices of *Abi3^−/−^* mice by 2.2- and 1.5-fold compared to *Abi3^+/+^* mice, respectively (Fig. 1, E and F). Furthermore, soluble Aβ40 and Aβ42 levels were increased by 1.8- and 1.6-fold in female *Abi3^−/−^* mouse cortices compared to *Abi3^+/+^* mice, respectively (Fig. 1, G and H).

We also determined the levels of insoluble Aβ40 and Aβ42 in the hippocampus using the same Aβ electrochemiluminescence method. Both Aβ40 and Aβ42 levels were increased in the hippocampi of male *Abi3^−/−^* mice by 1.5- and 1.4-fold compared to *Abi3^+/+^* mice, respectively (Fig. 1, I and J). Similarly, female *Abi3^−/−^* mice had 1.2-fold higher Aβ40 and Aβ42 levels in the hippocampus (Fig. 1, K and L). Moreover, there was a strong correlation between insoluble Aβ40 and Aβ42 levels in the cortices and hippocampi of these mice (fig. S3, A to D), validating the rigor of our biochemical assays.

### Increase of amyloid plaque load in *Abi3^−/−^* mice

**Fig. 2.**
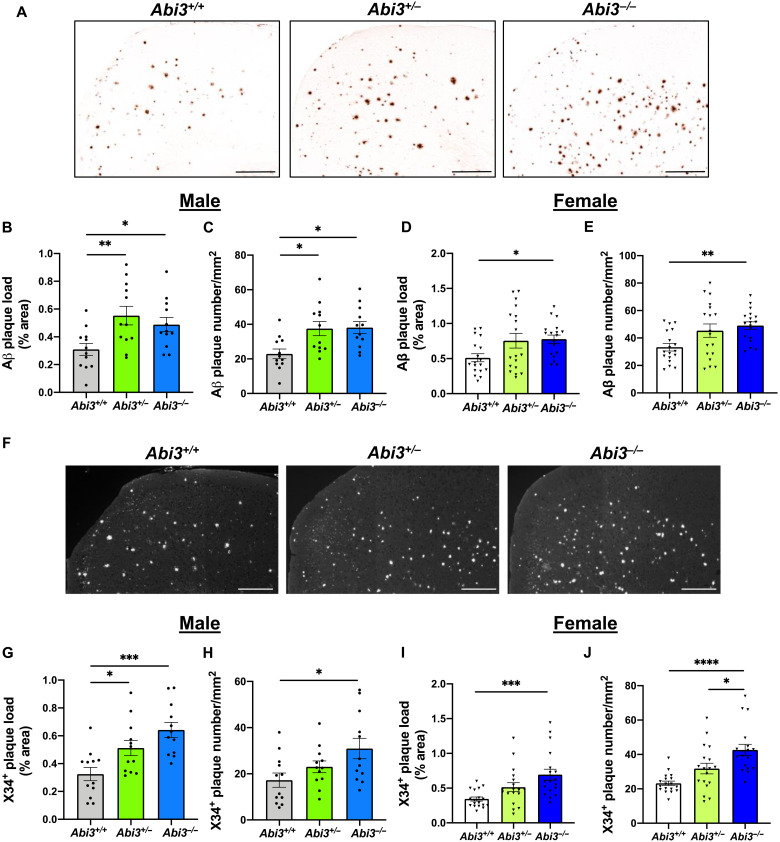
Deletion of *Abi3* gene locus increases amyloid plaques in 5XFAD mouse cortices. (**A**) Representative images showing coronal brain sections from 8-month-old *Abi3^+/+^*, *Abi3^+/−^*, and *Abi3^−/−^* mice stained with Aβ-specific 82E1 antibody. Scale bars, 300 μm. (**B**) Quantification of 82E1-positive Aβ plaque area and (**C**) the number of Aβ plaques in the cortices of male mice (*n* = 12 mice per genotype). (**D**) Quantification of 82E1-positive Aβ plaque area and (**E**) the number of Aβ plaques in the cortices of female mice (*n* = 18 mice per genotype). (**F**) Representative images of brain sections from 8-month-old *Abi3^+/+^*, *Abi3^+/−^*, and *Abi3^−/−^* mice stained with X34 dye that detects fibrillar plaques. Scale bars, 200 μm. The images are converted to grayscale, and white dots represent X34^+^ plaques. (**G**) Quantification of X34^+^ fibrillar plaque area and (**H**) the number of the plaques in male mouse cortices (*n* = 12 mice per genotype). (**I**) Quantification of X34^+^ fibrillar plaque area and (**J**) the number of the plaques in female mouse cortices (*n* = 18 mice per genotype). Data represent means ± SEM. One-way ANOVA and Tukey’s multiple comparisons test; *P < 0.05, **P < 0.01, ***P < 0.001, and ****P < 0.0001.

We further assessed amyloid plaque deposition by immunostaining the brain sections with anti-Aβ 82E1 antibody, which detects both diffuse and fibrillar Aβ (*26*) (Fig. 2A). Consistent with Aβ electrochemiluminescence assay results (Fig. 1), the extent of amyloid plaque accumulation was significantly increased in the cortices of male *Abi3^+/−^* and *Abi3^−/−^* mice (Fig. 2, B and C). Deletion of *Abi3* locus exacerbated Aβ accumulation also in female 5XFAD mouse cortices (Fig. 2, D and E). To characterize the nature of aggregates in the plaques, we stained the brain sections with X34 dye that detects only fibrillar amyloid deposits (Fig. 2F). We detected a significant increase in fibrillar amyloid plaque load in the cortices of male (Fig. 2, G and H) and female *Abi3^+/−^* and *Abi3^−/−^* mice (Fig. 2, I and J). Together, these data demonstrate that deletion of *Abi3* locus aggravates Aβ aggregation in a mouse model of Aβ amyloidosis.

To further understand the molecular mechanisms by which the deletion of *Abi3* locus increases amyloid accumulation, we assessed the levels of proteins involved in Aβ synthesis and degradation (fig. S4). Unexpectedly, we detected an increase in amyloid precursor protein (APP) and the β-C-terminal fragment (β-CTF) of APP by 1.7- and 2.5-fold in *Abi3^−/−^* mice compared to *Abi3^+/+^* mice, respectively (fig. S4, A to C). There was no change in the levels of β-secretase 1 (BACE-1), the enzyme that cleaves APP and generates β-CTF, between the groups (fig. S4D). To determine whether deletion of *Abi3* locus impairs Aβ degradation, thereby increasing the levels of Aβ, we assessed the levels of Aβ-degrading enzymes. The levels of insulin degrading enzyme (IDE) and neprilysin (NEP), two major Aβ-degrading enzymes, did not differ between the genotypes (fig. S4, E to G).

The increase in APP level can be the underlying mechanism of Aβ increase in *Abi3^−/−^* mice. However, these data were obtained from brains of 8-month-old 5XFAD mice when there was already a significant amount of Aβ load and accompanying pathologies. Because of the advanced pathologies at this old age, it is not easy to determine whether the increase in APP level could be the initial trigger of the increase in amyloid level or simply a response to the worsened pathology. To determine whether the increase in APP level can be the initial driver of the increased Aβ levels in *Abi3^−/−^* mice, it will be ideal to use younger mice with less amyloid level. To address this critical question, we generated a younger cohort of mice. We measured the levels of APP at an earlier age (4.5 months old) when the Aβ pathology was much less compared to 8 months of age (fig. S5A). Unlike the 8-month-old group, there was no significant difference in APP levels between the genotypes in the young cohort (fig. S5B). We further assessed the levels of other proteins involved in Aβ production and degradation in the young cohort. Similarly, there was no difference in BACE-1, β-CTF, IDE, and NEP protein levels between *Abi3^+/+^* and *Abi3^−/−^* mice (fig. S5, C to G). However, insoluble Aβ40 and Aβ42 levels were significantly higher in *Abi3^−/−^* mice compared to *Abi3^+/+^* mice (fig. S5, H and I) although APP levels did not differ between the genotypes in the young cohort. These data suggest that the increase in APP level in the old cohort may not be the initial driver of the increased Aβ deposition in *Abi3^−/−^* mice.

### Plaque-associated microglia are decreased in *Abi3^−/−^* mice

Because ABI3 is a microglia-enriched protein and microgliosis is one of the prominent features of AD pathology, we investigated whether ABI3 affects this phenotype in 5XFAD mice. We performed ionized calcium binding adapter molecule 1 (Iba1) immunoblotting to assess the extent of microgliosis in these mice. Iba1 levels did not differ between the genotypes, suggesting that *Abi3* deletion may not alter overall microgliosis (fig. S6, A and B).

**Fig. 3.**
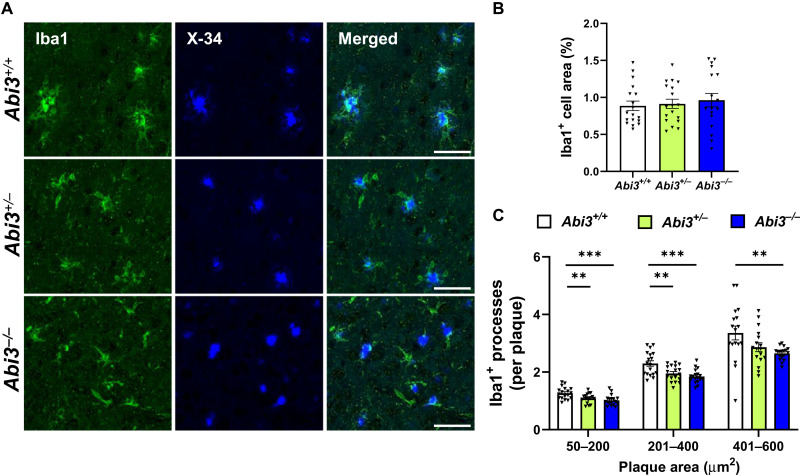
Deletion of *Abi3* locus decreases the number of plaque-associated microglia. (**A**) Representative images showing coronal brain sections stained with the microglial marker Iba1 antibody (green) and X34 fluorescent dye (blue). Scale bars, 50 μm. (**B**) The percent of area covered by Iba1 staining was quantified in the cortices of female mice. Area covered by Iba1^+^ cells did not differ between the genotypes. (**C**) The number of Iba1^+^ cells overlapping with X34^+^ plaques was quantified based on the size of each plaque. In each group, the number of Iba1^+^ cells per plaque was decreased in *Abi3^−/−^* mice compared to *Abi3^+/+^* mice (*n* = 18 per genotype). Data represent means ± SEM. One-way ANOVA and Tukey’s multiple comparisons test; **P < 0.01 and ***P < 0.001. See also fig. S6.

Although the role of microglia in AD is still debated, many studies suggest that microglia accumulate around Aβ plaques and clear Aβ deposits in transgenic mouse models of AD (*27*, *28*). To assess whether deletion of *Abi3* locus can alter the localization of microglia around plaques, we costained the brain sections with Iba1 antibody and X34 dye to detect myeloid cells, including microglia, and fibrillar plaques, respectively (Fig. 3A). Consistent with immunoblot results (fig. S6, A and B), the area covered by Iba1^+^ cells in the overall cortex was not different between the genotypes (Fig. 3B). These data imply that the deletion of *Abi3* locus may not affect the overall proliferation or survival of myeloid cells. However, the number of Iba1^+^ cells around X34^+^ plaques was significantly decreased in *Abi3^+/−^* and *Abi3^−/−^* mice (Fig. 3C). These data suggest that deletion of *Abi3* locus may prevent microglia from clustering around plaques, subsequently impairing Aβ clearance.

### Knockdown of *ABI3* gene expression impairs migration of microglia

**Fig. 4.**
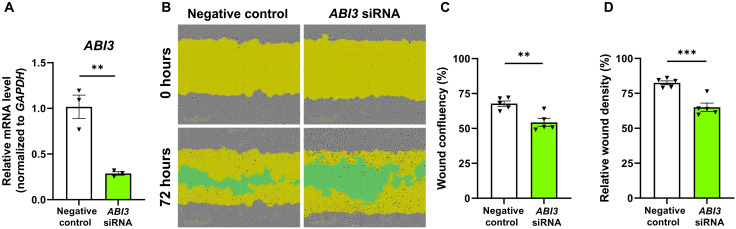
Knockdown of *ABI3* decreases migration of microglia in vitro. (**A**) HMC3 microglia cells were transfected with *ABI3*-targeting or negative control siRNAs. *ABI3* siRNA decreased mRNA level of *ABI3* by 72% in HMC3 cells (*n* = 3 per group). (**B**) A scratch was made in the middle of the wells with WoundMaker tool. Cell confluency within the scratch area was analyzed 72 hours after using the IncuCyte S3. Yellow area shows initial scratch area, and green area shows the scratch area without cells. (**C**) Wound confluency was decreased in ABI3 knockdown group. (**D**) Relative wound density was decreased in ABI3-silenced microglia (*n* = 5 per group). Data represent means ± SEM. Unpaired *t* test; **P < 0.01 and ***P < 0.001.

ABI3 is a component of WAVE regulatory complex that can regulate migration. Because we detected less microglia around amyloid plaques in *Abi3^−/−^* mice, we hypothesized that ABI3 may affect migration. To assess this, we knocked down *ABI3* gene in human HMC3 microglia cell line using a small interfering RNA (siRNA) approach and performed scratch-wound assay (Fig. 4, A and B). Silencing *ABI3* gene expression impaired migration of microglia as evidenced by reduced cell confluency in the wound area (Fig. 4C). Moreover, relative wound density, a measurement of cell confluency in the wound area normalized by the number of the cells outside of the scratch area, was lower in *ABI3*-silenced microglia (Fig. 4D). This suggests that the lower cell confluency in the scratch area is due to impaired migration, not proliferation, in the *ABI3* knockdown group.

### ABI3 regulates immune response

To gain more insight into the potential pathways that are regulated by *Abi3* in AD, we performed gene expression analysis using the Mouse AD Consortium panel (nCounter NanoString). This panel consists of 760 genes identified by the Accelerating Medicines Partnership Alzheimer’s Disease Project Consortium as LOAD relevant genes (*29*). We extracted RNA from cortices of male *Abi3^+/+^* and *Abi3^−/−^* mice for transcriptomic analyses. We used *P* < 0.05 as a cutoff to determine the differentially expressed genes (DEGs).

**Fig. 5.**
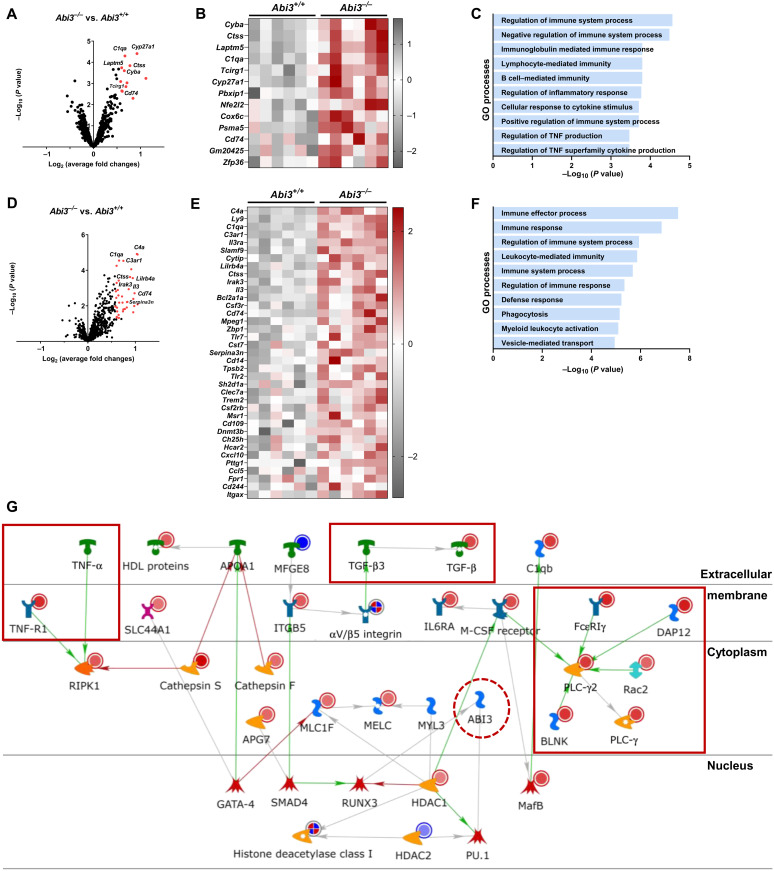
Deletion of *Abi3* locus alters immune response genes in 5XFAD mice. DEGs were identified in the cortices of 8-month-old male *Abi3^−/−^* mice compared to *Abi3^+/+^* mice using the nCounter NanoString mouse (**A** to **C**) AD panel and (**D** to **F**) Neuroinflammation panel (*n* = 6 per genotype). Volcano plots (A and D) demonstrate the fold change (*x* axis) and statistical significance level expressed as the −log_10_ *P* value (*y* axis). The red dots represent genes up-regulated significantly more than 1.5-fold in *Abi3^−/−^* mice compared to *Abi3^+/+^* mice. Heatmaps demonstrate the DEGs up-regulated more than 1.5-fold in *Abi3^−/−^* mice compared to *Abi3^+/+^* mice using the (B) AD and (E) Neuroinflammation panels. Gene ontology (GO) analyses were performed for DEGs in the (C) AD panel and (F) Neuroinflammation panel using the MetaCore software. (**G**) Pathmap analysis was performed using MetaCore. Up-regulated genes in our dataset are shown with red circles, and down-regulated genes are shown with blue circles in the pathmap. Green arrows between nodes represent activation, while gray arrows represent interaction with no specific direction of effect. Enriched genes involved in inflammatory processes are shown in red rectangles. See also fig. S7. IL6RA, interleukin-6 receptor α; HDL, high-density lipoprotein; M-CSF, macrophage colony-stimulating factor; TNF, tumor necrosis factor; PLC-γ, phospholipase C–γ; APG7, autophagy related 7; APOA1, apolipoprotein A1; BLNK, B cell linker; C1qb, complement C1q B chain; DAP12, TYRO protein tyrosine kinase-binding protein; GATA-4, GATA binding protein 4; HDAC1, histone deacetylase 1; ITGB5, integrin subunit beta 5; MafB, MAF BZIP transcription factor B; MELC, myosin essential light chain; Rac1, Rac family small GTPase 1; RIPK1, receptor-interacting serine/threonine-protein kinase 1; RUNX3, RUNX family transcription factor 3; SLC44A1, solute carrier 44A1; SMAD4, SMAD family member 4.

Among the DEGs, *C1qa* (complement system), *Cyp27a1* (lipid metabolism), *Ctss* (autophagy), *Laptm5* (autophagy), and *Cyba* (axon, dendrite structure) were the most up-regulated genes in *Abi3^−/−^* mice (Fig. 5, A and B, and table S2). To understand the specific biological pathways that are regulated by DEGs in *Abi3^−/−^* mice, we performed pathway enrichment analysis using MetaCore. Gene ontology (GO) analysis highlights enrichment of immune response–related pathways for the DEGs (Fig. 5C). Regulation of these microglial functions by *Abi3* may have contributed to the increase of Aβ accumulation in *Abi3^−/−^* mice.

Because *Abi3* is a microglia-enriched gene and pathway enrichment analysis from the Mouse AD Consortium panel suggests immune-related functions for ABI3, we further analyzed these samples using the Mouse Neuroinflammation panel (nCounter NanoString) to gain insight into the immune-related pathways. Consistent with the AD Consortium panel data, this panel also identified *C1qa* and *Ctss* as one of the most significantly up-regulated genes in *Abi3^−/−^* mice (Fig. 5, D and E). In addition, other complement pathway genes (*C4a* and *C3ar1*) and immune response genes (*Lilrb4a*, *Cd74*, *Il-3*, and *Tlr7*) were up-regulated in *Abi3^−/−^* mice (Fig. 5, D and E, and table S3). We observed an increase in *Serpina3n*, a gene that is known to be associated with AD risk (*30*), in *Abi3^−/−^* mice. GO analysis of the DEGs within this panel also highlights inflammatory response (*C1qa*, *C4a*, *C4b*, *Ctss*, *Lilrb4a*, *Cd74*, *Fcer1g*, and *Irak1*) pathways (Fig. 5F). Pathmap analysis using results from both AD Consortium and Neuroinflammation panels identified enrichment of immune response, complement system, and cytoskeleton pathways (Fig. 5G and fig. S7). Network analysis further supported these findings by identifying the genes that are involved in immune response and cytoskeleton networks (Table 1). Together, our data demonstrate that the deletion of *Abi3* locus elicits transcriptional changes in the genes related to key microglia functions, such as complement activation and immune signaling.

**Table 1.** Network analysis of differentially expressed genes (DEGs) in *Abi3^−/−^* mice. DEGs were identified in the cortices of 8-month-old male *Abi3^−/−^* mice compared to *Abi3^+/+^* mice using nCounter NanoString mouse AD and Neuroinflammation panels (*n* = 6 per genotype). Network analysis was performed using MetaCore. Enriched networks and the genes that are differentially regulated in *Abi3^−/−^* mice in each network are listed in the table.

**Networks**	***P* value**	**Network objects**
Immune response_Phagosome in antigen presentation	3.092 × 10^−4^	FcγRI, VAV-1, MSN (moesin), Btk, PSMB2, CD74, FCGR3A, Rac1, β-2-macroglobulin (B2M), Gelsolin, A2M, Cathepsin S, BLNK, FcγRIIα, FcεRIγ, and PLC-γ2
Immune response_Phagocytosis	1.124 × 10^−3^	FcγRI, VAV-1, MSN, Btk, FCGR3A, Rac1, APOE, PKC-ε, Gelsolin, Adenosine A2a receptor, p22-phox, FcγRIIβ, RelA (p65 NF-κB subunit), BLNK, FcγRIIα, FcεRIγ, and PLC-γ2
Cardiac development_BMP TGFβ signaling	3.864 × 10^−3^	TGF-β receptor type II, E2F1, TGF-β receptor type I, Rb protein, PKC-ε, TGF-β1, BMPR1B
Proliferation_Negative regulation of cell proliferation	5.921 × 10^−3^	MDM2, TGF-β receptor type II, Securin, STAT1, TGF-β receptor type I, Rb protein, Chk1, TGF-β1, OSMR, and IL6RA
Cytoskeleton_Macropinocytosis and its regulation	1.720 × 10^−2^	VAV-1, Rab-7, Rac1, SNX1, and M-CSF receptor
Cytoskeleton_Actin filaments	1.728 × 10^−2^	MSN, ARPC1B, Utrophin, Rac1, Gelsolin, and c-Abl
Translation_Selenium pathway	2.434 × 10^−2^	E2F1, RelA (p65 NF-κB subunit), and NRF2
Inflammation_IgE signaling	3.070 × 10^−2^	VAV-1, Btk, Rac1, PGDS, BLNK, FcεRIγ, and PLC-γ2
Immune response_Antigen presentation	3.130 × 10^−2^	FcγRI, TNF-R1, PSMB2, CD74, STAT1, B2M, Cathepsin S, and FcεRIγ
Development_EMT_Regulation of epithelial-to-mesenchymal transition	3.439 × 10^−2^	SIP1 (ZFHX1B), TGF-β receptor type II, TNF-R1, STAT1, DOCK2, Rac1, TGF-β receptor type I, TGF-β1, RelA(p65 NF-κB subunit), and c-Abl

**Fig. 6.**
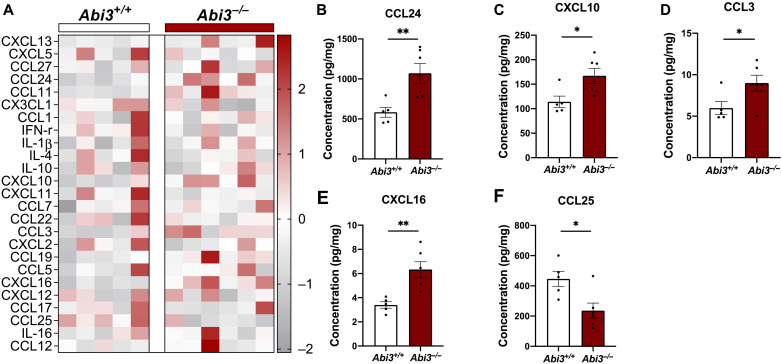
Deletion of *Abi3* locus alters the levels of immune response proteins in 5XFAD mice. Protein levels of chemokines and cytokines were measured in the PBS fraction of 8-month-old male 5XFAD mouse cortices using a 33-plex Bio-plex Pro mouse chemokine panel. (**A**) Among 33 proteins, 25 chemokines and cytokines were detected. Their differential protein levels are shown in the heatmap graph. (**B** to **F**) Significantly altered chemokines are shown as individual bar graphs. (B) CCL24, (C) CXCL10, (D) CCL3, and (E) CXCL16 were significantly increased, and (F) CCL25 was decreased in *Abi3^−/−^* mice compared to *Abi3^+/+^* mice. All data were normalized by total protein level and given as means ± SEM. Unpaired *t* test; *P < 0.05 and **P < 0.01 (*Abi3^+/+^*, *n* = 5; *Abi3^−/−^*, *n* = 6). IFN-r, interferon receptor; IL, interleukin.

Because neuroinflammation is one of the pathological hallmarks of AD and transcriptomic analyses suggest immune regulatory functions for the *Abi3* gene, we next assessed the protein levels of several inflammatory mediators in *Abi3^+/+^* and *Abi3^−/−^* mouse brains. Chemokines regulate migration of immune cells (e.g., monocytes and microglia) to the site of inflammation in neurodegenerative diseases, including AD (*31*). Moreover, Aβ can induce cytokine release from microglia, hence aggravating the pathology (*32*). Therefore, we determined the protein levels of secreted cytokines and chemokines in cortex using the Bio-Plex Pro Mouse Chemokine Assay. This assay can detect 33 chemokines and cytokines, of which we were able to detect 25 in the brain. The levels of several chemokines were significantly altered in *Abi3^−/−^* mouse brain (Fig. 6A). Among these, CCL24, CXCL10, CCL3, and CXCL16 were increased, whereas CCL25 was significantly decreased in *Abi3^−/−^* mice compared to *Abi3^+/+^* mice (Fig. 6, B to F). These data provide yet another layer of evidence supporting the role of ABI3 in brain inflammation. *Ccl3* was identified as one of the common top hits in microglial transcriptome profiling from aging, amyloidosis, and tauopathy models (*33*). We demonstrated an increase in CCL3 protein level in *Abi3^−/−^* mouse brains. Therefore, it is still possible that ABI3 may affect different mechanisms in addition to amyloid pathology.

Next, we assessed phagocytic activity of microglia after silencing *Abi3* gene expression (fig. S8). Because HMC3 cells show low phagocytic activity for pHrodo-labeled zymosan, we used BV2 mouse microglia cells. We confirmed knockdown of *Abi3* gene expression in BV2 cells by measuring *Abi3* mRNA level after siRNA treatment (fig. S8A). Knockdown of *Abi3* expression significantly reduced engulfment of zymosan (fig. S8, B and C). To assess whether knockdown of *Abi3* also affects Aβ uptake, we treated BV2 cells with oligomeric Aβ42 and measured intracellular Aβ levels. There was no significant difference in the levels of intracellular Aβ42 in the *Abi3* siRNA-treated group compared to negative control (fig. S8D). This difference between zymosan and Aβ uptake may arise from the differences in the uptake mechanisms for zymosan and Aβ. To phagocytose Aβ plaques, microglia need to migrate toward them. Because we detected less microglia clusters around Aβ plaques in *Abi3^−/−^* mice (Fig. 3C) and reduced migration in *ABI3* knockdown cells (Fig. 4), the impaired migration of microglia might be the major driver of the increased Aβ accumulation in *Abi3^−/−^* mice (Figs. 1 and 2).

### Synaptic function is impaired in *Abi3^−/−^* mice

**Fig. 7.**
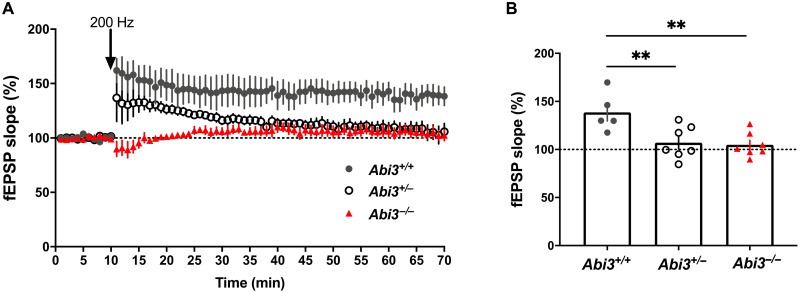
Deletion of *Abi3* locus alters synaptic functions in 5XFAD mice. (**A**) *Abi3* deficiency reduced LTP in male 5XFAD mice. (**B**) Data show average of normalized fEPSP slope for the final 10 min of recording (60 to 70 min) relative to 10 min baseline average. Data analyzed using one-way ANOVA and Dunnett’s multiple comparisons test; *n* = 5 slices from four *Abi3^+/+^* mice, *n* = 7 slices from six *Abi3^+/−^* mice, and *n* = 7 slices from six *Abi3^−/−^* mice. Data represent means ± SEM. **P < 0.01.

Synaptic failure is one of the major pathophysiological changes observed in patients with AD and several transgenic mouse models of AD. It is considered an early time point molecular change that is followed by neuronal loss and cognitive deficits (*1*, *34*). Numerous in vitro and in vivo studies have demonstrated that Aβ can deteriorate synaptic functions and block long-term potentiation (LTP), which is the cellular basis of learning and memory (*35*). Because we detected increase in Aβ accumulation in *Abi3^−/−^* mice, we decided to assess synaptic functions in our mouse models. We performed LTP experiments on brain slices from 6-month-old male mice. Field excitatory postsynaptic potentials (fEPSPs) were recorded from the CA1 region of the hippocampus and LTP was induced using high-frequency stimulation. LTP was significantly decreased in *Abi3^+/−^* and *Abi3^−/−^* mice compared to *Abi3^+/+^* mice (Fig. 7, A and B). This finding suggests that deletion of *Abi3* locus impairs synaptic function in 5XFAD mice.

### Immune gene–targeted single-cell transcriptomic analysis identified distinct microglia subclusters in *Abi3^−/−^* mice

**Fig. 8.**
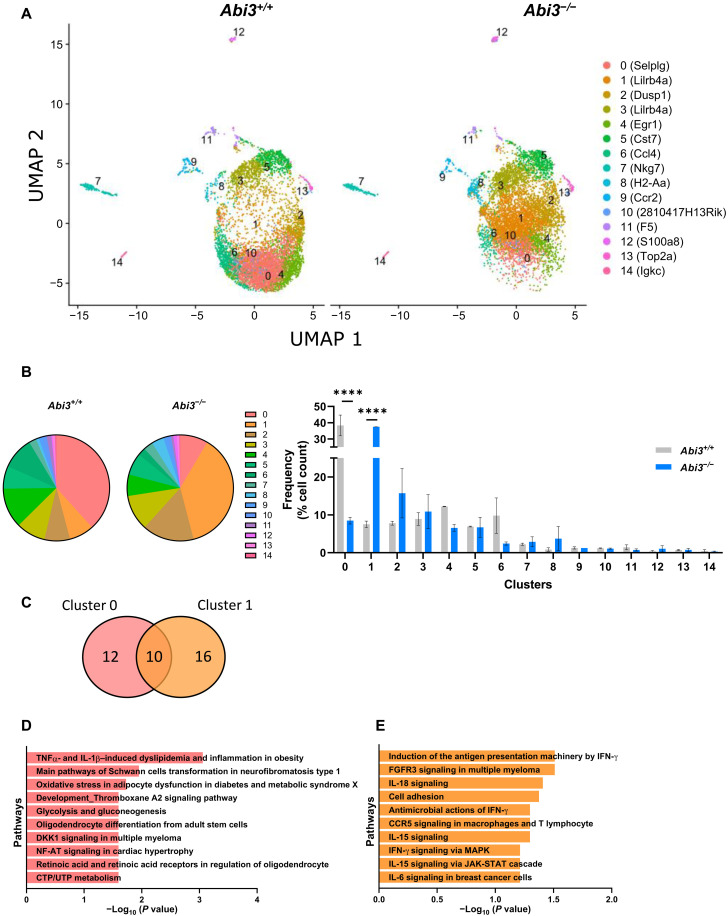
Targeted scRNA-seq identifies a distinct microglia signature in *Abi3^−/−^* mice on 5XFAD background. (**A**) Uniform Manifold Approximation and Projection (UMAP) dimensionality reduction plot showing 14 clusters of cells derived from 9-month-old male *Abi3^+/+^* and *Abi3^−/−^* mouse brain (*Abi3^+/+^*, *n* = 6970 cells; *Abi3^−/−^*, *n* = 6799 cells). Top marker genes (as ranked by significance) are displayed after each cluster number in the legend. (**B**) Percentage of cell populations in each cluster was compared between the genotypes. Cluster 0 is the major cell population in *Abi3^+/+^* mice, whereas cluster 1 is the major cell population in *Abi3^−/−^* mice (left). While the cells in cluster 0 significantly decreased, cluster 1 population significantly increased in *Abi3^−/−^* mice compared to *Abi3^+/+^* mice (right). Data represent means ± SEM. Two-way ANOVA and Sidak’s multiple comparisons test; ****P < 0.0001. (**C**) The number of genes detected in clusters 0 and 1. (**D** and **E**) Pathway analyses were performed using the MetaCore software. Pathway analysis of (D) cluster 0–specific genes and (E) cluster 1–specific genes. CTP/UTP, cytidine 5′-triphosphate/uridine 5′-triphosphate; IFN-γ, interferon-γ; JAK-STAT, Janus kinase signal transducers and activators of transcription; MAPK, mitogen-activated protein kinase; FGFR3, fibroblast growth factor receptor 3; NF-AT, nuclear factor of activated T-cells.

Recent advancements in scRNA-seq technology enabled in-detail trancriptomic analysis of different cell types and identified unique cellular signatures, specifically in microglia, in AD (*36*, *37*). Because *Abi3* is enriched in microglia and our data demonstrate that deletion of *Abi3* locus markedly aggravates AD pathology, we hypothesized that *Abi3* deletion can alter microglial transcriptomic signatures in an Aβ amyloidosis mouse model. To test this hypothesis, we performed scRNA-seq on *Abi3^+/+^* and *Abi3^−/−^* mouse brains using the BD Rhapsody Mouse Immune Response Panel (Fig. 8). This panel enabled us to compare 397 immune-related genes at a single-cell level in *Abi3^+/+^* and *Abi3^−/−^* mouse brains (table S4). To enrich glial cells, we used a gentle enzymatic dissocation method that we optimized previously (*38*). The commonly used single-cell isolation methods performed above 4°C are known to trigger aberrant activation of cells (*39*). Therefore, we performed all steps, including an enzymatic digestion step, at 4°C. Unsupervised clustering revealed 14 distinguished clusters across the samples (Fig. 8A). These clusters were identified based on the expression of known cell type–specific markers: microglia (clusters 0 to 6 and 10), T cells (cluster 7), macrophages (clusters 8 and 9), neutrophils (cluster 12), and B cells (cluster 14) (Fig. 8A and table S5) (*40*, *41*). Clusters 11 and 13 could not be assigned to any specific cell type due to having a limited number of marker genes in the panel. However, we were able to identify many different microglia clusters due to the higher sensitivity of the targeted scRNA-seq approach compared to the whole-transcriptome approach. We identified significant differences in the proportion of cells in clusters 0 and 1 microglia between *Abi3^+/+^* and *Abi3^−/−^* mice. While cluster 0 constitutes most of the cells in *Abi3^+/+^* mice, cluster 1 is the major cell population in *Abi3^−/−^* mice (Fig. 8B). There was a significant shift in cell populations between cluster 0 (38.34% in *Abi3^+/+^* and 8.47% in *Abi3^−/−^*) and cluster 1 (7.52% in *Abi3^+/+^* and 37.47% in *Abi3^−/−^*) owing to the deletion of *Abi3* locus (Fig. 8B). To identify the pathways that are regulated by cluster 0 and cluster 1 genes, we performed pathway analyses using genes specific to cluster 0 or cluster 1 microglia (Fig. 8C and table S6). While cluster 0 genes are mainly involved in metabolism-related pathways, cluster 1 genes are involved in immune response pathways (Fig. 8, D and E). Enrichment of cluster 1 genes in *Abi3^−/−^* mice further supports the role of ABI3 in neuroinflammation.

**Fig. 9.**
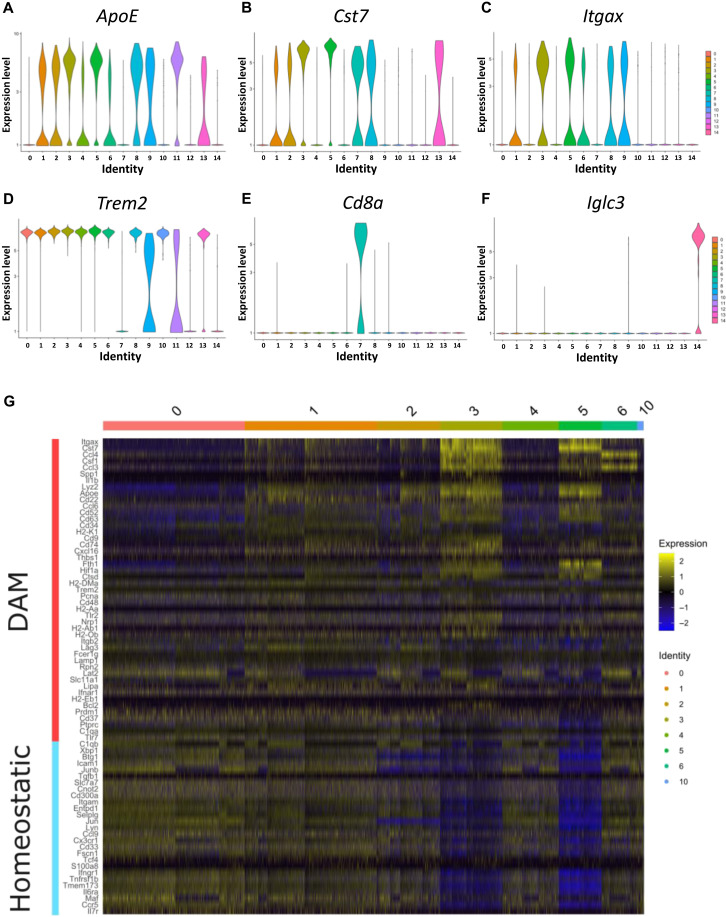
Expression of DAM genes and other cell type–specific marker genes in scRNA-seq analysis. (**A** to **F**) Targeted scRNA-seq analysis identified 14 clusters of cells derived from 9-month-old male *Abi3*^+/+^ and *Abi3*^−/−^ mouse brain (*Abi3^+/+^*, *n* = 6970 cells; *Abi3^−/−^*, *n* = 6799 cells). Each plot displays the raw counts on the *y* axis and cluster identity on the *x* axis. Among the DAM marker genes, the expression of (A) *Apoe*, (B) *Cst7*, (C) *Itgax*, and (D) *Trem2* were analyzed for each cluster. (E) *Cd8a*, T cell marker gene, was detected only in cluster 7. (F) *Iglc3*, B cell marker gene, was detected only in cluster 14. (**G**) Heatmap showing DAM and homeostatic microglia marker genes across microglial clusters (0 to 6 and 10). The level of gene expression is averaged per gene across all clusters; up-regulation compared to the mean expression level is shown in yellow and down-regulation in blue.

Single-cell transcriptomic analyses identified enrichment of a unique microglia type, disease-associated microglia (DAM), in 5XFAD mouse model (*36*). These cells demonstrate increased expression of several AD risk genes, such as *ApoE*, *Trem2*, *Tyrobp*, and *Lpl*. On the other side, the expression of homeostatic microglia marker genes, including *Cx3cr1*, *Tmem119*, and *P2ry12*, decreases in DAM cells. To determine whether *Abi3^−/−^* mice demonstrate these microglia phenotypes, we analyzed the levels of DAM marker genes for each cluster in our dataset (Fig. 9, A to D). DAM marker genes were initially identified in 5XFAD mice when 5XFAD mice were compared to wild-type mice (*36*). In our data, these markers were detected in most microglia clusters because all mice used for our scRNA-seq experiment were already on 5XFAD background. Among the DAM genes, *Apoe*, *Cst7*, and *Itgax* were more represented in clusters 3 and 5, attributing a more DAM signature to these microglia clusters (Fig. 9, A to C). We also confirmed the specificity of the marker genes by detecting *Cd8*, a T cell marker gene, and *Iglc3*, a B cell marker gene, in relevant clusters 7 and 14, respectively (Fig. 9, E and F). To more thoroughly characterize microglia subpopulations in our dataset, we used scRNA-seq data from 5XFAD mice (*36*) and compared the microglia clusters (clusters 0 to 6 and 10) in our dataset with their DAM and homeostatic microglia populations. We were able to detect 47 DAM and 27 homeostatic marker genes using the BD Rhapsody Mouse Immune Response Panel (Fig. 9G). Although we cannot directly compare our targeted scRNA-seq data with the whole transcriptome approach owing to technical variables (sensitivity, read count depth per gene, etc.), we found that clusters 3 and 5 exhibited gene expression patterns similar to DAM phenotype (Fig. 9G). Because we identified a significant shift in the proportion of cells belonging in clusters 0 and 1 in *Abi3^−/−^* mice compared to *Abi3^+/+^* mice (Fig. 8B), we investigated whether this shift in cluster proportion reflects a shift in DAM or homeostatic microglia phenotypes. As all our experimental mice carry the 5XFAD transgene, both clusters 0 and 1 represent DAM phenotype to some extent (Fig. 9, A to D). However, cluster 1 appears to be more DAM-like compared to cluster 0 (Fig. 9G). Because cluster 1 microglia proportion increased in *Abi3^−/−^* mice (Fig. 8B), the genotype with more Aβ accumulation, these data suggest that DAM phenotype is more represented in *Abi3^−/−^* mice.

Although our immune targeted scRNA-seq approach provides a higher sensitivity, it has a limited number of genes in the panel. Therefore, it is still possible that deletion of *Abi3* locus can alter other genes that were not available in the targeted scRNA-seq panel. In an attempt to explore this possibility, we performed bulk RNA-seq experiments to identify other pathways regulated by the deletion of *Abi3* locus. We found marked down-regulation of several genes, including *Gm11942*, *Gm17511*, *Gngt2*, *Gm43903*, *Gm10039*, and *Gm26793* in *Abi3^−/−^* mice compared to *Abi3^+/+^* mice (fig. S9A). Pathway analysis demonstrated the enrichment of cytoskeleton remodeling and immune response (fig. S9B), consistent with our NanoString transcriptomic analyses (Table 1).

## DISCUSSION

In recent years, human genetics studies of AD have significantly contributed to our understanding of the disease by identifying many risk genes. For example, a rare coding missense variant in the *ABI3* gene is associated with an increased risk of AD (*8*, *9*). However, functional validation studies are necessary to establish the causality of these genetic risk factors and to dissect out the pathways regulated by these genes. To determine the role of ABI3 in the pathogenesis of AD, we examined how the loss of *Abi3* function affects pathological features of AD using the 5XFAD mouse model. We demonstrated that *Abi3* deficiency markedly increased soluble and insoluble Aβ levels and amyloid plaque load both in male and female mice. Consistent with its microglia specific gene expression pattern (*8*, *9*, *11*), we found that deletion of *Abi3* locus altered the expression of genes regulating microglial phagocytosis and immune response. Together, our data suggest that, among multiple pathways by which ABI3 may affect the pathogenesis of AD, Aβ accumulation and neuroinflammation are the main underlying mechanisms.

Microglia migrate toward amyloid plaques, phagocytose Aβ, and degrade it (*28*). Because this mechanism is known to play a critical role in Aβ clearance, deficits in any of these steps can result in amyloid buildup in AD brains. It has recently been shown that microglial surveillance is dependent on actin polymerization (*42*). Moreover, phagocytic response requires cytoskeletal rearrangements for microglia to engulf Aβ (*43*). Because ABI3 is involved in actin regulation (*13*, *44*), loss of *Abi3* gene may impair actin remodeling and thereby migration of microglia toward Aβ plaques. Supporting this hypothesis, we detected less microglia surrounding the plaques in *Abi3^−/−^* mice, similar to what has been observed in *Trem2* knockout (*Trem2^−/−^*) mice (*45*, *46*). Furthermore, we demonstrated that knocking down *Abi3* expression with siRNA reduced the migration of microglia cells, suggesting that this mechanism may underlie the increased Aβ accumulation in *Abi3^−/−^* mice.

In addition to Aβ aggregation, AD is also characterized by neuroinflammation. In support of this, recent AD genetic studies have demonstrated that immune and microglia networks are critical players in AD (*3*, *4*). Our transcriptomic and gene enrichment analyses identified the up-regulation of inflammatory genes in *Abi3^−/−^* mice. This finding is further supported by elevated chemokine protein levels in *Abi3^−/−^* mice. Because inflammatory proteins (e.g., interleukin-1β, tumor necrosis factor–α, and interferon-γ) can alter APP expression, APP processing, or Aβ transport (*47*–*50*), the inflammatory changes caused by the deletion of *Abi3* locus may have contributed to the increase in APP protein level and subsequently Aβ accumulation. Although the alterations in the neuroinflammatory response may exacerbate the progression of AD, whether these are the direct consequences of loss of *Abi3* gene or a secondary consequence triggered by the increase in Aβ accumulation warrants further investigation.

Based on our findings in NanoString transcriptomic analyses and the predicted role for ABI3 in the immune system, we conducted scRNA-seq analysis specifically focusing on immune genes. By using a targeted assay, we were able to detect different types of immune cells and the notable changes in these subpopulations in the brain due to the deletion of *Abi3* locus. We identified that cluster 0 (*Selplg*-high) and cluster 1 (*Lilrb4a*-high) microglia subpopulations were markedly altered in *Abi3^−/−^* mice. Although these changes in microglia subpopulations can be direct consequences of *Abi3* loss, it is also possible that such changes are secondary responses to the *Abi3*-mediated changes in the pathology. For example, Aβ oligomers and fibrils can induce microglial activation, which subsequently increases the expression of inflammatory genes in microglia (*51*). Furthermore, recent scRNA-seq studies identified marked transcriptional changes in microglia in response to Aβ (*52*). Because we detected a significant increase in Aβ accumulation in *Abi3^−/−^* mice, microglial transcriptomic changes that we identified in scRNA-seq analysis could be secondary to the increased Aβ pathology in *Abi3^−/−^* mice.

Because our novel targeted scRNA-seq approach has a high sensitivity, it enabled us to capture the small changes in immune cell populations, which can be lost in whole transcriptome scRNA-seq assays due to its low sensitivity. Thus, the targeted approach reduced the need for high sequencing depth and subsequently decreased the sequencing cost. Because our Immune Response scRNA-seq platform has only 397 genes in the panel, it is likely that we failed to detect transcriptomic changes in other cell types, caused by the deletion of *Abi3* locus. However, our whole transcriptome bulk RNA-seq data also suggest that immune-related pathways are dysregulated in *Abi3^−/−^* mice. Notably, bulk RNA-seq data analysis identified marked down-regulation of several genes in *Abi3^−/−^* mice compared to *Abi3^+/+^* mice. Of these, *Gngt2* and *Gm10039* are located on the same chromosome with the *Abi3* gene. It is possible that the deletion of the *Abi3* locus may inadvertently disrupt the expression of other genes near or even far away from this locus as shown in multiple knockout mouse models, including a widely used *Trem2* knockout mouse model (*53*). This possibility might be especially high for the *Gngt2* gene because it is adjacent to the *Abi3* gene. However, we also cannot rule out the possibility that the deletion of *Abi3* gene can affect the expression of other genes that are indirectly regulated by ABI3 protein under physiological conditions. Because genes on different chromosomes, such as *Gm11942*, *Gm17511*, and *Gm43903*, are also notably down-regulated in *Abi3^−/−^* mice, these differential expressions might be due to the depletion of ABI3 protein in *Abi3^−/−^* mice. It is possible that these markedly dysregulated genes may also be contributing to the pathologies that we detected in *Abi3^−/−^* mice and warrant further investigation.

We identified similar phenotypic changes in *Abi3^−/−^* and *Trem2^−/−^* mice. Although the effect of *Trem2* deletion on Aβ burden varies depending on the disease stage and mouse model (*20*–*23*), many studies demonstrated that deletion of *Trem2* impairs microglia clustering around Aβ plaques (*45*, *46*). Furthermore, lack of *Trem2* reduces chemotaxis and phagocytosis of microglia (*54*, *55*). It was also demonstrated that *Trem2* activates multiple signaling pathways involved in actin cytoskeleton organization (*54*). Therefore, it is tempting to speculate that *Trem2* may be an upstream regulator of *Abi3*. However, this warrants further studies.

Last, we observed a significant LTP impairment in *Abi3*-deficient 5XFAD mice. Three mechanisms may contribute to this synaptic impairment. First, this might be due to an increase in immune response in *Abi3^−/−^* mice as demonstrated by transcriptomic and biochemical analyses. Increased inflammatory cytokines are known to impair LTP in the hippocampus, affecting synaptic plasticity (*56*). Specifically, CXCL10 and CCL3, which were increased in *Abi3^−/−^* mice, were found to be elevated in patients with AD and associated with neurological deficits (*57*–*60*). Both CXCL10 and CCL3 have been shown to impair LTP and synaptic function (*61*, *62*). Second, ABI3 may directly affect synapses by modulating actin remodeling. Although *ABI3* is mainly expressed in microglia, its expression in neurons and interaction with filamentous actin (F-actin) in dendritic spines were observed in primary neuronal cell culture (*44*, *63*). Overexpression and down-regulation of *Abi3* altered spine morphology and synaptic density (*63*). These findings strengthen the potential role of ABI3 in synapse function. Last, Aβ can induce neurotoxicity through various mediators, such as inflammatory molecules, membrane lipids, and cytoskeletal proteins (*35*). Moreover, even low levels of Aβ can facilitate synaptic F-actin depolymerization and impair dendritic spines at very early stages of the pathology in an APP/presenilin 1 transgenic mouse model (*64*). Therefore, LTP impairment might be due to an increase in Aβ levels in *Abi3*-deficient mice. Further investigation is warranted to identify the exact mechanism by which *Abi3* regulates synaptic function.

We have demonstrated that Aβ levels were significantly increased when *Abi3* locus was deleted before amyloid accumulation. However, such data do not necessarily mean that decreasing ABI3 protein level after amyloid accumulation will have the same effect. Because AD GWAS variants are identified based on their association with AD risk rather than the progression of AD, it remains unknown how such variants may affect the disease progression, which is more important for the development of treatment. This concept is even more critical for targeting microglia genes because microglia may respond differently to AD pathology, depending on the stage of the disease.

Together, we demonstrated that the deletion of *Abi3* locus significantly exacerbates several key features of AD pathology. In future studies, it is warranted to investigate the functional consequences of the *ABI3* S209P risk variant on AD pathology using animal models with the S209P mutation in the *ABI3* gene. Drug targets based on human genetics data are known to double the success rate in clinical development (*65*). Given our functional data shown in this study and its significant genetic association with AD (*8*–*10*), ABI3 and its downstream pathways may be promising therapeutic targets.

## MATERIALS AND METHODS

### Experimental design

This study was designed to investigate the role of *Abi3* gene in the pathogenesis of AD. To accomplish this aim, we generated a mouse model of Aβ amyloidosis, lacking the *Abi3* gene. We evaluated the effects of *Abi3* deletion on Aβ levels, amyloid plaques, neuroinflammation, and synaptic function using enzyme-linked immunosorbent assay, immunohistochemistry, Western blot, and electrophysiology techniques. Sample sizes were determined based on the statistical power calculations from our experiences and other studies in the literature (*26*, *66*, *67*). Using an SD of 400, an effect size of 40% for insoluble Aβ42 levels, a power of 0.8, and *P* < 0.05, we aimed to generate at least 12 mice per sex for each genotype. The order of animals was randomized for each experiment. The researchers were blinded during the quantification of histology samples. Figure legends contain sample sizes and statistical tests used.

### Animals

5XFAD mice (*66*) were purchased from the Jackson Laboratory [Mutant Mouse Resource & Research Centers (MMRRC) 34840, B6SJL-Tg(APPSwFlLon,PSEN1*M146L*L286V)6799Vas/Mmjax)], and *Abi3^−/−^* mice were obtained from the MMRRC, University of California, Davis, Mouse Biology Program [C57BL/6N-Abi3tm1.1(KOMP)Vlcg]. The *Abi3^−/−^* mouse strain used for this study was generated using the Velocigene “definitive null” targeting strategy. To ensure the deletion of *Abi3* gene, a vector targeted the *Abi3* gene locus on chromosome 11 from 95,842,143 to 95,832,627. To determine the gene dosage effect of *Abi3* on amyloid deposition, 5XFAD mice were crossed with *Abi3^−/−^* mice to generate 5XFAD mice expressing two copies of *Abi3* (*Abi3^+/+^*), one copy of *Abi3* (*Abi3^+/−^*), and knockout for *Abi3* (*Abi3^−/−^*). We used both male and female mice and analyzed the results separately owing to sex-dependent difference in the rate of amyloid accumulation in 5XFAD mice. Because SJL genetic background carries the *Trem2^S148E^* allele, a naturally occurring variant, we genotyped all the experimental mice for this variant. All mice were heterozygous for this single-nucleotide polymorphism (SNP), except two female mice in the *Abi3^+/−^* group. Although it is not known whether this SNP has any functional consequences, to be on the safe side, we excluded these two homozygous mice from all analyses. Mice were housed under standard conditions with free access to food and water. Eight- or 4.5-month-old mice were used for biochemical experiments. We used 6-month-old mice for electrophysiology and 9-month-old mice for scRNA-seq experiments. All animal experiments were approved and performed in compliance with the guidelines of the Institutional Animal Care and Use Committee at Indiana University.

### Human postmortem brain tissue

Human postmortem brain tissues were provided by the Alzheimer’s Disease Research Center at Icahn School of Medicine at Mount Sinai, with well-characterized criteria for clinical and pathological diagnoses. The proteome dataset generated in Bai *et al.* (*18*) was used to analyze the protein level of ABI3 in cognitively normal and LOAD patient brains.

### Tissue collection and sample preparation

Mice were deeply anesthetized with Avertin (250 mg/kg, intraperitoneal) and transcardially perfused with cold phosphate-buffered saline (PBS). Brains were immediately removed, and the left hemisphere was fixed in 4% paraformaldehyde for 24 hours at 4°C to be used in histology. Tissue samples were embedded in paraffin and sectioned at the Histology and Histomorphometry Core, Indiana Center for Musculoskeletal Health, IUSM. Five-micrometer-thick coronal sections were transferred onto microscope slides and stored at room temperature. The right hemisphere was dissected into cortex and hippocampus and immediately frozen in dry ice. Samples were stored at −80°C until further processing for biochemical experiments.

### Western blotting

Proteins were sequentially extracted from brain tissues with PBS, radioimmunoprecipitation assay (RIPA), and 5 M guanidine buffer in the presence of protease and phosphatase inhibitors as we described previously (*68*). The RIPA fractions were used for the Western blot analysis. Equal amounts of proteins (20 μg) were loaded onto 4 to 20% TGX gels (Bio-Rad), separated by gel electrophoresis, and transferred onto polyvinylidene difluoride membranes. Blots were probed with the APP (1:1000; Invitrogen, 51-2700), BACE-1 (1:1000; Cell Signaling Technology, 5606), anti-Aβ 82E1 (1:1000; IBL-AMERICA, IBL10323), IDE (1:1000; Abcam, ab32216), NEP (1:1000; Abcam, 208778), Iba1 (1:1000; Abcam, ab178846), and β-actin (1:20,000; Sigma-Aldrich, A1978) antibodies. Signals were visualized by chemiluminescence [ECL Select (GE Healthcare) or TMA-6 ECL detection kit (Lumigen)]. Blots were quantified by densitometry with ImageJ software [National Institutes of Health (NIH)]. Results were normalized by β-actin levels and given as a fold change relative to *Abi3^+/+^* genotype.

### Electrochemiluminescence assay for Aβ detection

For Aβ40 and Aβ42 detection, V-PLEX Plus Aβ Peptide Panel 1 (6E10) Kit (Meso Scale Discovery (MSD), K15200E] was used. Soluble and insoluble Aβ40 and Aβ42 levels were measured using the PBS and guanidine extracts of the mouse samples following the manufacturer’s instructions, respectively. For Aβ uptake assay, RIPA extracts of BV2 cell lysates were used to measure levels of intracellular Aβ42. Signals were measured on a MESO QuickPlex SQ 120 (multiplexing imager, MSD).

For cytokine and chemokine measurement, PBS-soluble extracts from *Abi3^+/+^* and *Abi3^−/−^* male mouse cortices were used. Samples were loaded into Bio-Plex Pro Mouse Chemokine Panel (Bio-Rad, 12002231) to measure the levels of 33 chemokines. Cytokine and chemokine levels were determined using the Bio-Rad Bio-Plex 200 multiplex assay system. We were able to detect 25 of them in the brain samples. The concentrations were normalized by total protein levels in the samples.

### Immunohistochemistry

Coronal sections were deparaffinized, and antigen retrieval was performed with citrate buffer (10 mM, pH 6.0) in a microwave. For 3,3′-diaminobenzidine (DAB) staining, slides were treated with 0.3% H_2_O_2_ and blocked with tris-buffered saline containing 3% milk and 0.25% Triton X-100 at room temperature for 30 min. Sections were then incubated with anti-Aβ 82E1 antibody (1:500; IBL-AMERICA, IBL10323) in blocking solution at 4°C overnight and then with biotinylated goat anti-mouse secondary antibody (1:400, Thermo Fisher Scientific) at room temperature for 1 hour. Antibody binding was detected with Vectastain ABC Elite (Vector Laboratories, PK6100) and DAB peroxidase (horseradish peroxidase) substrate kits (Vector Laboratories, SK-4100) according to the manufacturer’s instructions. Sections were dehydrated with increasing concentrations of ethanol (50 to 95%), cleared with xylene, and coverslipped with mounting medium (Permount, Thermo Fisher Scientific).

For fibrillar plaque staining, we used X-34 dye that detects β sheet structure proteins. Sections were permeabilized with 0.25% Triton X-100 in PBS at room temperature for 30 min on a shaker. Subsequently, slides were incubated with 0.01 mM X-34 in staining buffer (40% ethanol and 0.02 N NaOH, in PBS) for 20 min. Sections were then rinsed with washing buffer (40% ethanol in PBS) and PBS before coverslips were applied with Aqua-Poly/Mount mounting medium.

For immunofluorescence staining, sections were stained with X-34 and then blocked with PBS containing 10% normal donkey serum at room temperature for 1 hour, incubated with anti-Iba1 (rabbit polyclonal, 1:1000, Abcam) in blocking solution at 4°C overnight, and then incubated with Alexa Fluor 488–donkey anti-rabbit immunoglobulin G (1:1000, Invitrogen) at room temperature for 1 hour. Sections were coverslipped with Aqua-Poly/Mount mounting medium.

### Quantification of immunohistochemistry data

Images were captured on a digital pathology slide Scanner (Aperio VERSA; Leica Biosystems). Staining was quantified in cortical region using Fiji (ImageJ, NIH) (*69*) and CellProfiler 3.1.9 (Broad Institute) (*70*). The average of three sections from different anatomical coordinates (150 μm distant) was used to represent plaque or Iba1^+^ cell load for each mouse. The number of plaques was normalized by total area of analyzed region.

### Migration assay

The human microglia cell line (HMC3) was cultured in Dulbecco’s modified Eagle’s medium (DMEM) + GlutaMAX (Gibco, 10566016), supplemented with 10% fetal bovine serum (FBS) (Gibco, 16000) and 1% penicillin-streptomycin (Gibco, 15140). siGENOME ABI3-targeting and negative control nontargeting siRNAs were purchased from Dharmacon. Cells were seeded in a 96-well plate and transfected with 40 nM *ABI3* siRNA or negative control siRNA. Twenty-four hours after transfection, a scratch was made in the middle of the wells with the WoundMaker tool (Essen Bioscience, 4563) and imaged for 72 hours using the IncuCyte S3 live-cell imager (Essen Bioscience). Cell migration was assessed by measuring the wound confluency and relative wound density using the IncuCyte software. Wound confluency is the confluency of cells within the wound region. Relative wound density is the measure of cell density in the wound area relative to the cell density outside of the wound area.

### Phagocytosis assay

BV2 mouse microglia cells were cultured in DMEM, supplemented with 2% FBS and 1% penicillin-streptomycin. Cells were seeded in a 24-well plate and transfected with *Abi3*-targeting or negative control siRNAs. Forty-eight hours after transfection, cells were treated with pHrodo-labeled zymosan (50 μg/ml; Essen Bioscience, 4617) for 4 hours, and nuclei were stained with Hoechst (4 μg/ml). The fluorescent intensity within the cells was measured using the ArrayScan XTI (Thermo Fisher Scientific) imaging system scanning at ×10 magnification.

### Aβ uptake assay

Oligomeric Aβ42 was prepared as described before (*71*). Briefly, Aβ42 peptide powders were dissolved in hexafluoroisopropanol (HFIP) (Sigma-Aldrich, 52517) to remove preexisting aggregates. HFIP was evaporated in a fume hood overnight, followed by drying down in SpeedVac for 1 hour. The resulting peptide films were dissolved in dimethyl sulfoxide to prepare 5 mM stocks, sonicated in a bath sonicator for 10 min, and diluted to 100 μM in phenol-red free DMEM/F12 medium (Thermo Fisher Scientific, 21-041-025). To prepare Aβ oligomers, the stocks were incubated at 4°C for 24 hours. BV2 cells were seeded in a 24-well plate and transfected with *Abi3*-targeting or negative control siRNAs. Twenty-four hours after transfection, cells were treated with 200 nM oligomeric Aβ42 for 1 hour. The proteins were extracted using RIPA buffer, including protease and phosphatase inhibitors, and sonicated in a bath sonicator. The homogenates were centrifuged at 17,000*g* for 15 min and the supernatants were collected for Aβ measurement. Total protein amount was measured by bicinchoninic acid assay and intracellular Aβ levels were normalized by total protein amount for each sample.

### Proteomics analysis

The proteome dataset generated in Bai *et al.* (*18*) was used in this study. Briefly, human brain cortical tissues from control cases (*N* = 23) and patients with AD (*N* = 39) were lysed, and protein samples were labeled with Tandem-Mass-Tag (TMT) for whole proteome analysis as described previously (*18*). The samples were analyzed by liquid chromatography–tandem mass spectrometry. The analysis was performed using the JUMP software (*72*). Individual protein abundance was corrected by the cell-type composition using cell type–specific markers for neuron, astrocyte, microglia, and oligodendrocyte. The change of each cell type was estimated on the basis of protein abundance of the cell type markers. To correct for cell-type effect, log-transformed TMT intensity of each protein was fitted to a linear regression model against the estimated cell-type change and then adjusted as the residuals plus the intercept.

### Total RNA extraction and qPCR

Total RNA was extracted from cortical tissues using TRIzol (MRC). RNA concentration and quality were determined using the Nanodrop 2000 Spectrophotometer (Thermo Fisher Scientific). For real-time qPCR, mRNAs were reverse transcribed with a High Capacity cDNA Reverse Transcription kit (Applied Biosystems). qPCR was performed in QuantStudio 3 using the default thermal cycling condition with Power SYBR (Applied Biosystems) with the following primers: *Abi3* forward primer, CTACTGCGAGGATAACTACTTGC; and reverse primer, CAGGTTACCCACTTGGTAGGC. Mouse *Gapdh* endogenous control was used as a normalization reference. Relative mRNA levels were calculated by comparative cycle threshold method.

### NanoString analyses

Cortical regions of 8-month-old male *Abi3^+/+^* and *Abi3^−/−^* mice were used for transcriptomic analysis. The NanoString Mouse AD and Mouse Neuroinflammation gene expression panels were used for gene expression profiling on the nCounter platform (NanoString, Seattle, WA) as described by the manufacturer. Total RNA was diluted to 15 ng/μl for these experiments. To construct linear models for the effects of *Abi3* gene deletion on transcriptome, Limma version 3.44.3 was run in RStudio version 1.3.959 using R version 4.0 on log-normalized NanoString panel gene expression data. Pathway and network analyses were done using the MetaCore software.

### Bulk RNA-seq library preparation and sequencing

Isolated RNA was shipped to Lexogen Inc., Austria, for library preparation and sequencing. Libraries were prepared manually according to the manufacturer’s instructions using QuantSeq 3′ mRNA-Seq FWD Library Prep Kit (Lexogen, UG, version 015UG009V0252). Briefly, 50 ng of total RNA was denatured for 3 min at 85°C with oligo(dT) primers containing an Illumina-compatible sequence at its 5′ end, followed by reverse transcription at 42°C for 15 min. After RNA removal, the second strand of the cDNA was synthesized by a random primer containing an Illumina-compatible linker sequence at its 5′ end, and the end product was purified by a magnetic bead-based method to remove all reaction components. Last, individual sample barcodes (dual indexing) for multiplexing were introduced via PCR (cycle number determined by qPCR), and the leftover reaction components were removed by a magnetic bead-based purification method. All libraries were analyzed for adapter dimers, size distribution, and concentration on a Fragment Analyzer System using the DNF-474 HS NGS Fragment kit (1 to 6000 base pairs) (Agilent). After pooling the libraries in an equimolar ratio, the concentration and the size distribution of the lane mix were analyzed by Qubit dsDNA HS assay (Thermo Fisher Scientific) and by 2100 Bioanalyzer device using the HS-DNA assay (Agilent), respectively. A 2 nM dilution of the lane mix was denatured and diluted to loading concentration for sequencing on a NextSeq 500 instrument with the SR75 High Output Kit (Illumina). Seventy-six–base pair single-end reads were generated at Lexogen Services.

### QuantSeq 3′ mRNA-Seq sequencing data processing

The FastQ files from the RNA-seq were processed on the basis of the workflow outlined in the Lexogen’s user guide “QuantSeq 3′ mRNA-Seq Integrated Data Analysis Pipelines on BlueBee Genomics Platform” (Lexogen, UG, version 015UG108V0310). First, adapter sequences were trimmed using bbduk tool from BBTools v.38.72 with the following parameters: *k = 13 ktrim = r useshortkmers = t mink = 5 qtrim = r trimq = 10 minlength = 20*. The reference FASTA file of adapters (*truseq_rna.fa* from the BBTools suite) and a FASTA file of poly(A) and poly(T) were used for trimming. The reference FASTA file for poly(A) and poly(T) was a string of 18 As and 18 Ts. FastQC v. 0.11.5 was used to determine the quality of trimmed reads. An indexed mouse reference genome using the Ensembl GRCm39 v.103 primary assembly was made using STAR v.2.7.3a with the parameters *–runMode genomeGenerate --genomeFastaFiles Mus_musculus.GRCm39.dna.primary_assembly.fa --sjdbGTFfile Mus_musculus.GRCm39.103.gtf --sjdbOverhang 75* (*73*). The trimmed reads were aligned to the indexed mouse reference genome using STAR v. 2.7.3a using the parameters *--outFilterType BySJout --outFilterMultimapNmax 20 --alignSJoverhangMin 8 --alignSJDBoverhangMin 1 --outFilterMismatchNmax 999 --outFilterMismatchNoverLmax 0.6 --alignIntronMin 20 --alignIntronMax 1000000 --alignMatesGapMax 1000000 --outSAMattributes NH HI NM MD --outSAMtype BAM SortedByCoordinate* (*74*). Gene read counts were obtained using the htseq-count tool of HTSeq v.0.9.1-py13 with Python v. 2.7.13 with the parameters *-m intersection-nonempty -s yes -f bam -r pos* (*75*).

### Differential gene expression analysis of bulk RNA-seq data

The gene read count files were imported into RStudio v.1.4.1103 running R v.4.0.4. Differential expression analysis was performed using DESeq2. The DESeq dataset was created using the *DESeqDataSetFromHTSeqCount* function with the design argument *~ Condition* (the *Abi3* genotype) and the reference level to *Abi3^+/+^* condition. Only genes with total read counts greater than or equal to 10 were included in the estimations of size factors, dispersion, and negative binomial generalized linear model fitting. Genes with average normalized read counts (base mean) greater than or equal to 1.5 were included in the results. DEGs were considered to be genes with a false discovery rate–adjusted (Benjamini-Hochberg) *P* value less than or equal to 0.05.

### Single-cell RNA library preparation

Nine-month-old male *Abi3^+/+^* and *Abi3^−/−^* mice were anesthetized and transcardially perfused with sterile PBS, and the brain was removed. The cerebellum was removed from the right hemisphere, which was immediately finely minced on ice and transferred to a polypropylene tube containing ice-cold Accutase (Gibco, A11105-01). Tissue was incubated with rocking at 4°C for 30 min and then pelleted through centrifugation at 300*g* for 5 min at 4°C. Supernatant was aspirated and samples were resuspended in ice-cold wash buffer [1× Hanks’ balanced salt solution (Gibco, 14175095) and 0.04% bovine serum albumin (BSA; Miltenyi Biotec, 130-091-376)] and gently dissociated through pipetting. After all samples were processed, the cloudy upper suspension was transferred to a fresh tube, and the remaining clumps of tissue were further dissociated using additional ice-cold wash buffer. Cell suspensions were pelleted through centrifugation at 300*g* for 10 min at 4°C. The supernatant was aspirated and cells were resuspended in degassed MACS Myelin Removal Buffer [1× DPS (Gibco 14190250) and 0.5% BSA (Miltenyi Biotec, 130-091-376)]. Myelin removal beads (Miltenyi Biotec, 130-096-733) were added to each sample and mixed thoroughly. Samples and beads were incubated for 15 min at 4°C with rotating, washed with myelin removal buffer, and then pelleted through centrifugation at 300*g* for 10 min at 4°C. Supernatant was aspirated and samples were resuspended in myelin removal buffer.

LS columns (Miltenyi Biotec, 130-042-401) were loaded into a QuadroMACS separator on a MACS MultiStand with Tube Rack (Miltenyi Biotec, 130-090-976, 130-042-303, and 130-091-052). Columns were equilibrated with myelin removal buffer. Cell suspensions were loaded onto columns, and myelin-depleted cell suspensions were washed through using myelin removal buffer. Cells were pelleted through centrifugation at 300*g* for 5 min at 4°C. Supernatant was aspirated and cells were gently resuspended using a pipette. Cell numbers and viability were quantified using Draq7 (BD Biosciences, 564904) and Calcein-AM (BD Biosciences, 564061) staining. BD Rhapsody Mouse Immune Response Panel (BD Biosciences, 633701 and 633753) was used for the targeted scRNA-seq library preparation. Single-cell RNA libraries were checked for quality using QuBit (Thermo Fisher Scientific) and TapeStation (Agilent) and then submitted to the Centre for Medical Genomics and sequenced on an Illumina NovaSeq 6000 SP (200 cycle kit) with 20% PhiX spike-in.

### scRNA-seq data analysis

FastQ files were uploaded to SevenBridges and processed using the BD Rhapsody Targeted Analysis Pipeline using default settings. DBEC-adjusted read counts were downloaded, imported into R version v4.0.2 in RStudio v1.3.959, and analyzed using Seurat v3.2.0 (*76*). Cells with fewer than 40 unique reads and more than 1.5× interquartile ranges from the mean (>59,300 total reads) were excluded from the analysis. Data were log-normalized, and cells were clustered using the first 10 principal components based on an Elbow Plot. Cluster marker genes were identified using the FindConservedMarkers function. The tissue dissociation protocol that we used is designed to enrich glial cells (*38*). We annotated the clusters by comparing the marker genes per cluster with scRNA-seq data from the brain-derived myeloid cell dataset of Li *et al.* (*40*). For the markers showing no association with cell types from this dataset, we then considered canonical markers, such as *Igkc* and *Cd19* (B cells), and *Cd8a* and *Trbc2* (T cells). For the remaining unannotated clusters, we considered data from the Mouse Cell Atlas v2.0 (*41*). Where most of the marker genes were not consistently associated with one cell lineage (e.g., clusters 11 and 13), cells were not annotated. Pathway analyses were performed using the MetaCore software.

### Electrophysiology

Six-month-old male *Abi3^+/+^*, *Abi3^+/−^*, and *Abi3^−/−^* mice were used for electrophysiology experiments. Mice were anesthetized with isoflurane and decapitated, and the brain was quickly removed. Sagittal slices (350 μm) were cut in a 95% O_2_ and 5% CO_2_-saturated, ice-cold sucrose-based solution (194 mM sucrose, 30 mM NaCl, 4.5 mM KCl, 1 mM MgCl_2_, 26 mM NaHCO_3_, 1.2 mM NaH_2_PO_4_, 10 mM glucose) using a Leica VT1200S vibratome. The slices were stored for 60 min in an artificial cerebral spinal fluid (aCSF) (124 mM NaCl, 4.5 mM KCl, 1 mM MgCl_2_, 26 mM NaHCO_3_, 1.2 mM NaH_2_PO_4_, 10 mM glucose, and 2 mM CaCl_2_), saturated with 95% O_2_ and 5% CO_2_ at 30°C, and then kept at room temperature until recording. The recordings were performed at 30° to 32°C in a chamber that was perfused with oxygenated aCSF at a rate of 1 to 2 ml/min. Picrotoxin (100 μM) was added to the medium to block γ-aminobutyric acid type A receptors. fEPSPs were recorded with micropipettes filled with 1 M NaCl using a Multiclamp 700B amplifier and Clampex software (Molecular Devices). Signals were low-pass–filtered at 2 kHz and digitized at 50 kHz. Tungsten stereotrodes (~1 megohm) were used to stimulate the Schaffer collaterals in the hippocampus CA1 region. Stimulation parameters were adjusted using a constant current isolated stimulator (Digitimer). Using the stimulation strength that produced 50% of the maximum intensity, a stable baseline was recorded for 10 min stimulating at 0.05 Hz. LTP was induced by applying 10 200-Hz trains of 100-ms duration every 5 s (*77*, *78*). Changes in the slope of the response (millivolts per millisecond) fEPSPs were recorded for 60 min after induction monitoring at 0.05 Hz. Data were expressed as a percentage of change with respect to the average baseline.

### Quantification and statistical analyses

Statistical analyses were performed using GraphPad Prism 8 (GraphPad Software). Multiple group analyses were performed by one-way analysis of variance (ANOVA) followed by Tukey’s post hoc test for normally distributed datasets. Plaque distribution analyses were performed by two-way ANOVA followed by Tukey’s post hoc test. The correlation between Aβ40 and Aβ42 levels was analyzed with Pearson’s correlation test. Electrophysiology data were analyzed using one-way ANOVA and Dunnett’s multiple comparisons test. Data were presented as means ± SEM. Sample sizes and statistical analyses for each experiment are indicated in figure legends.

## Notes

View/request a protocol for this paper from *Bio-protocol*.
