# Supplementary material for: Deletion of Abi3 gene locus exacerbates neuropathological features of Alzheimer’s disease in a mouse model of Aβ amyloidosis
Source: Sci Adv. 2021 Nov 3;7(45):eabe3954. doi: 10.1126/sciadv.abe3954 (PMC8565913; doi:10.1126/sciadv.abe3954)
Supplement: Supplementary file 1 — Figs. S1 to S9 [file sciadv.abe3954_sm.pdf]

Science Advances
AAAS

Supplementary Materials for

Deletion of *Abi3* gene locus exacerbates neuropathological features of Alzheimer's disease in a mouse model of Aβ amyloidosis

Hande Karahan, Daniel C. Smith, Byungwook Kim, Luke C. Dabin, Md Mamun Al-Amin, H. R. Sagara Wijeratne, Taylor Pennington, Gonzalo Viana di Prisco, Brianne McCord, Peter Bor-chian Lin, Yuxin Li, Junmin Peng, Adrian L. Oblak, Shaoyou Chu, Brady K. Atwood, Jungsu Kim*

*Corresponding author. Email: jk123@iu.edu

Published 3 November 2021, *Sci. Adv.* **7**, eabe3954 (2021)
DOI: 10.1126/sciadv.abe3954

The PDF file includes:

Figs. S1 to S9

Other Supplementary Materials for this manuscript include the following:

Tables S1 to S6

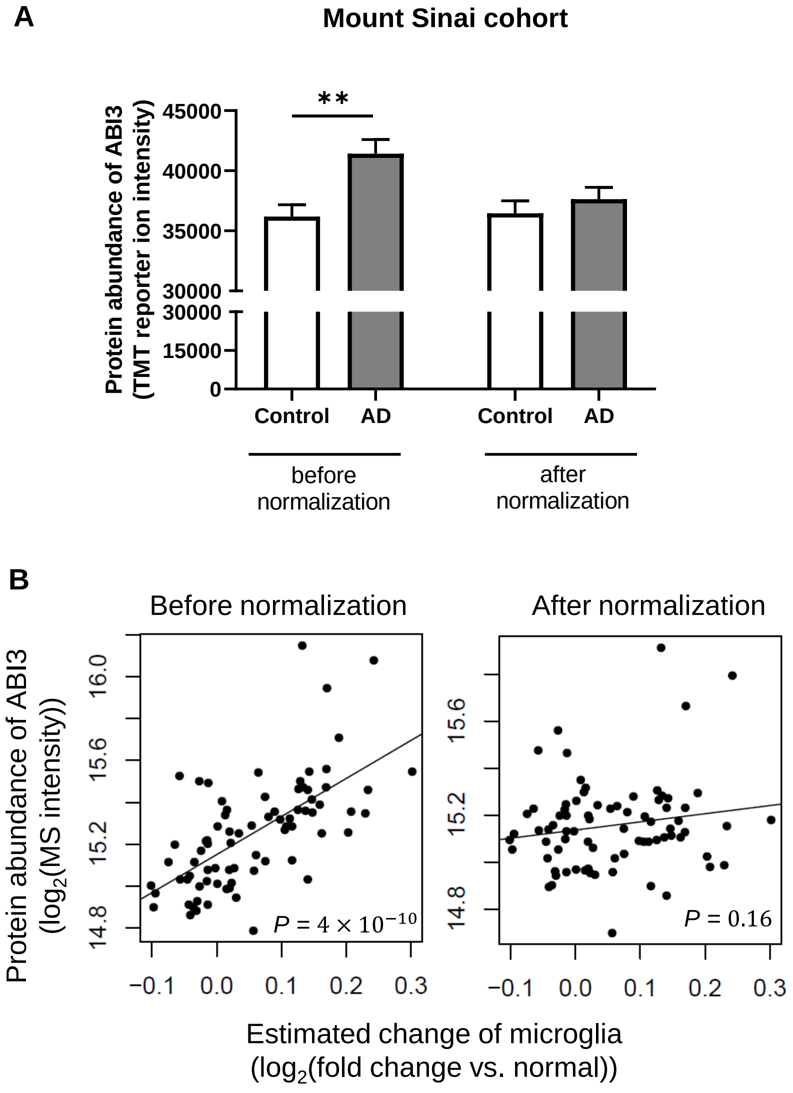

Fig. S1. ABI3 protein level in AD patient brains.

Mass spectrometry analysis were performed on parahippocampal cortices of LOAD patient and control group from the Mount Sinai cohort. **(A)** TMT intensities of ABI3 are shown. ABI3 protein level is significantly higher in AD patient brains before normalizing the data by cell type specific markers (left panel). The difference between two groups was not significant after normalizing the difference in the proportion of different cell types (right panel) (control, N=23; AD patients, N=39). Data represent mean ± SEM. Two-tailed unpaired *t* test; $**p<0.01$. **(B)** Correlation of the ABI3 protein intensity and microglia cell type before and after correction of a difference in cell type proportion.

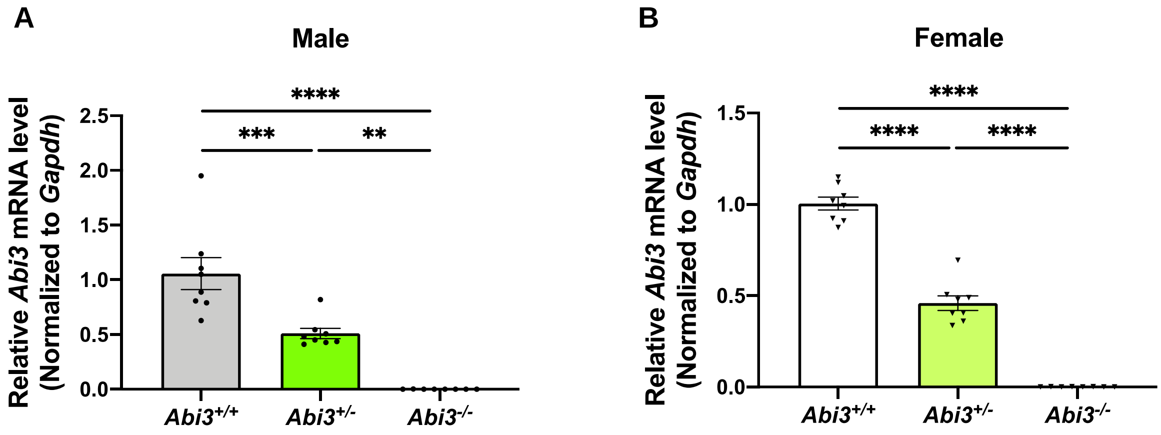

Fig. S2. Characterization of *Abi3* deficient 5XFAD mouse model.

Abi3 knock-out mice were bred with 5XFAD transgenic mouse model. *Abi3* mRNA levels were measured in the cortices of 8-month-old 5XFAD mice with two copies of *Abi3* (*Abi3*$^{+/+}$), one copy (*Abi3*$^{+/-}$), and no expression of *Abi3* (*Abi3*$^{-/-}$) by qPCR. **(A)** *Abi3* expression was decreased by 49% in male *Abi3*$^{+/-}$ mice and was not detected in *Abi3*$^{-/-}$ mice (n=8/genotype). **(B)** Similarly, *Abi3* expression was decreased in female *Abi3*$^{+/-}$ mice by 54% and was not detected in *Abi3*$^{-/-}$ mice (n=8/genotype). Data represent mean ± SEM. One-way ANOVA, Tukey's multiple comparison test; $^{**}p<0.01$, $^{***}p<0.001$, $^{****}p<0.0001$.

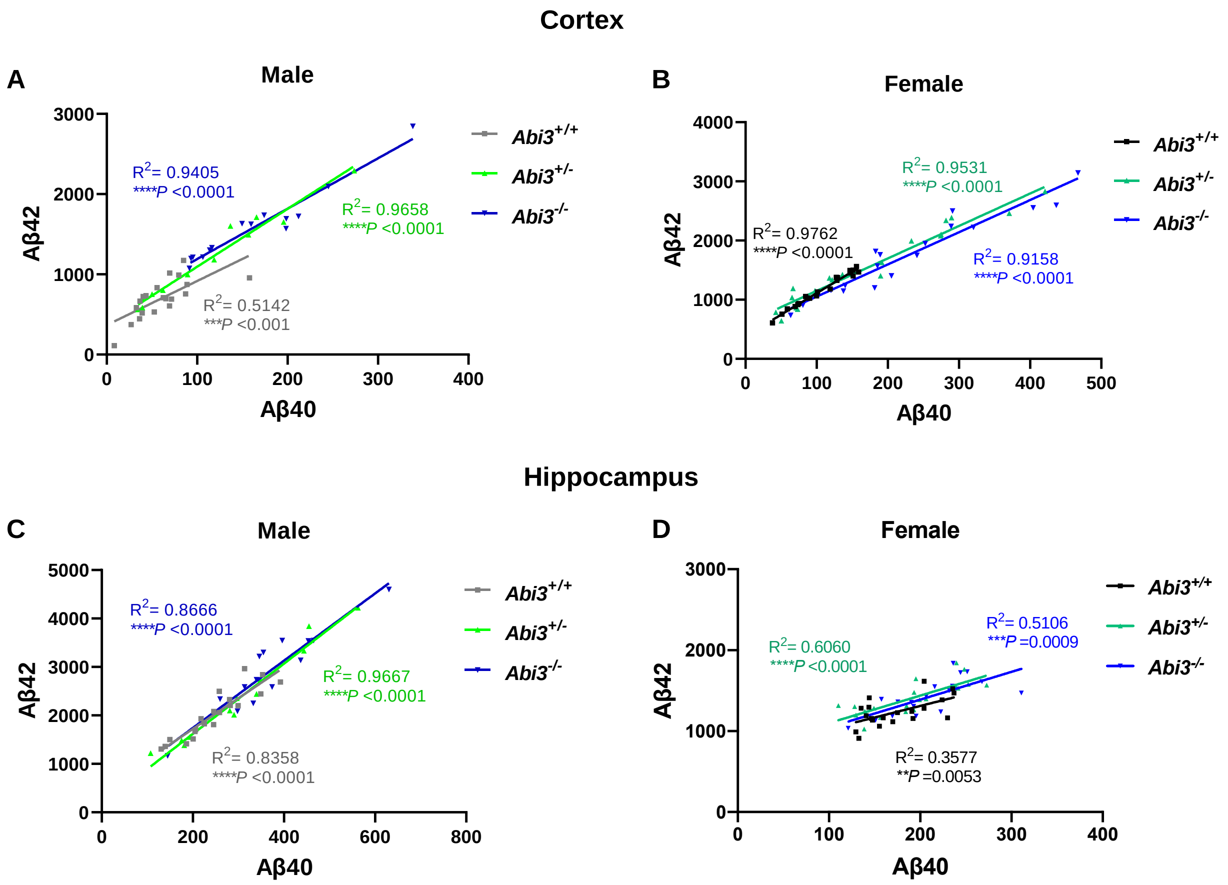

Fig. S3. The correlation between Aβ40 and Aβ42 levels.

(A to D) Insoluble Aβ40 and Aβ42 levels were measured in the guanidine fraction of 5XFAD mouse cortices using Meso Scale Discovery electrochemiluminescence assay (*Abi3*$^{+/+}$ n=20M, 20F; *Abi3*$^{+/-}$ n=12M, 20F; *Abi3*$^{-/-}$ n=14M,18F). Significant correlation was observed between insoluble Aβ40 and Aβ42 levels in the cortices of **(A)** male and **(B)** female mice. Each data point indicates an individual mouse. Correlation between Aβ40 and Aβ42 was analyzed in the hippocampus of **(C)** male and **(D)** female mice. Pearson's correlation analysis; ***p <0.001, ****p< 0.0001.

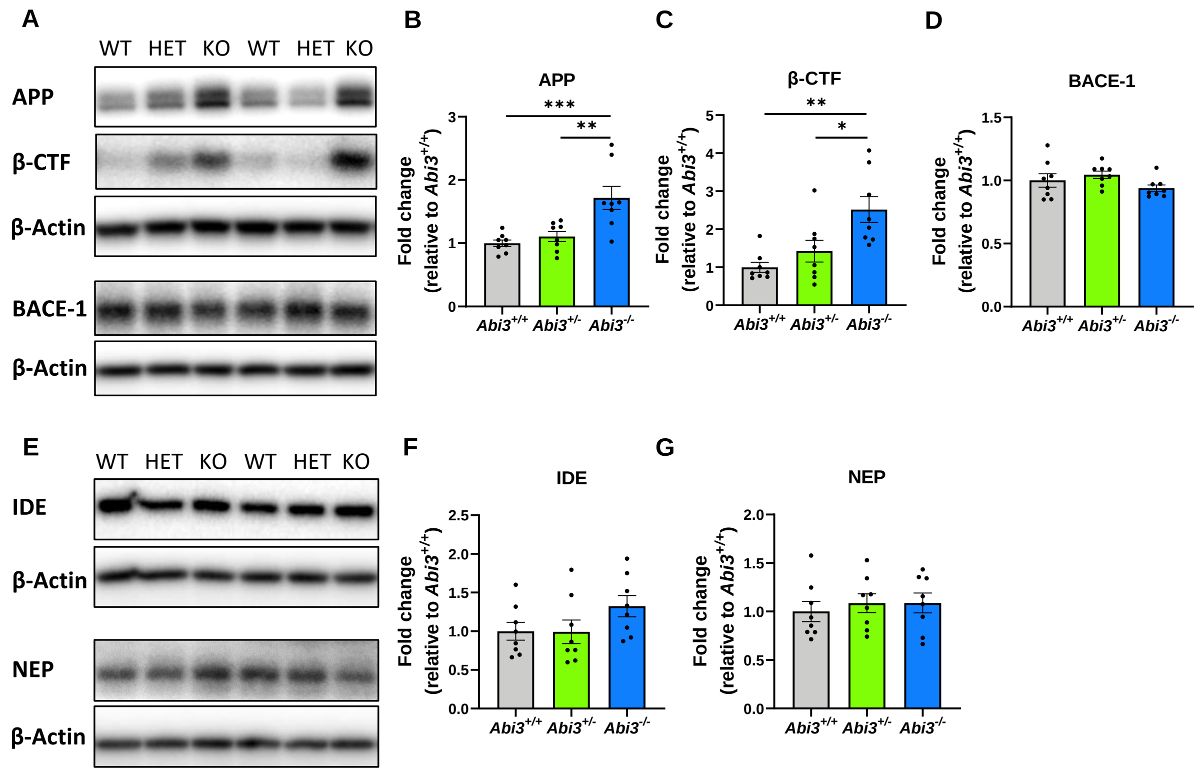

Fig. S4. Effect of *Abi3* locus deletion on Aβ production and degradation.

Protein levels in the RIPA fraction of male mouse cortices were determined by Western blot. **(A)** Representative images of the blots. Quantification of **(B)** APP, **(C)** β-CTF, and **(D)** BACE1 immunoblots. **(E)** Representative images of the blots. Quantification of **(F)** IDE and **(G)** NEP immunoblots. Data were normalized by β-actin levels and expressed as fold-change relative to *Abi3*$^{+/+}$ (n=8/genotype). Data represent mean ± SEM. One-way ANOVA, Tukey's multiple comparison test; *p <0.05, **p< 0.01, ***p< 0.001.

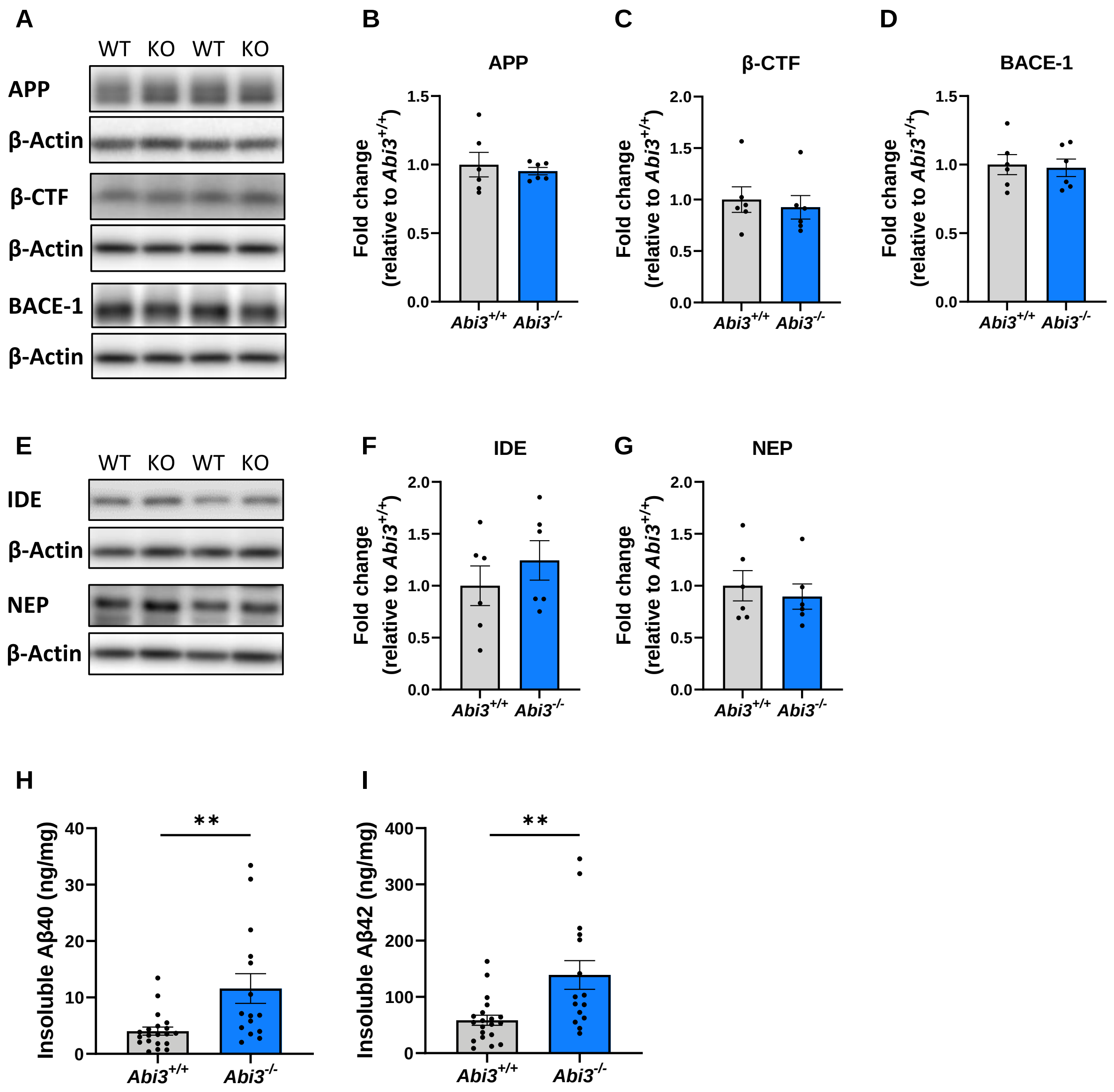

Fig. S5. Effect of *Abi3* locus deletion on APP processing and Aβ levels in the young cohort. **(A-I)** Protein levels in the RIPA fraction of 4.5-month-old male mouse cortices were determined by Western blot. **(A)** Representative images of the blots. Quantification of **(B)** APP, **(C)** β-CTF, and **(D)** BACE1 immunoblots. **(E)** Representative images of the blots. Quantification of **(F)** IDE and **(G)** NEP immunoblots. Data were normalized by β-actin levels and expressed as fold-change relative to *Abi3*$^{+/+}$ (n=6/genotype). Data represent mean ± SEM. Unpaired t-test; $p>0.05$. **(H-I)** Insoluble Aβ40 and Aβ42 levels were measured in the guanidine fraction of 5XFAD mouse cortices using Meso Scale Discovery electrochemiluminescence assay. Insoluble **(H)** Aβ40 and **(I)** Aβ42 levels were increased in the cortices of 4.5-month-old male *Abi3*$^{-/-}$ mice compared to *Abi3*$^{+/+}$ mice (*Abi3*$^{+/+}$ n=20, *Abi3*$^{-/-}$ n=15). Data represent mean ± SEM. Unpaired t-test; **p< 0.01.

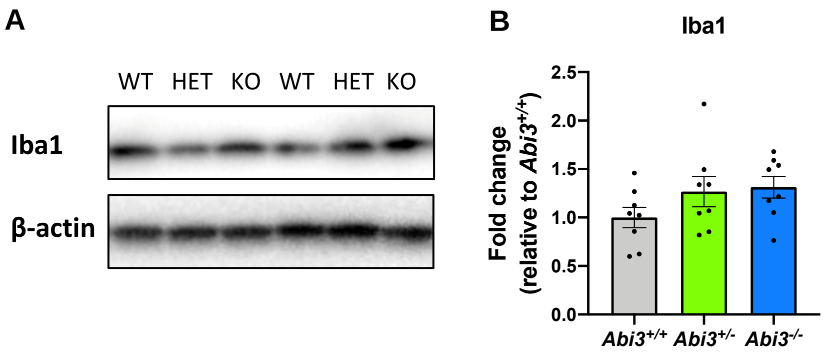

Fig. S6. Effect of *Abi3* locus deletion on microgliosis.

RIPA fraction of male cortical extracts were used for Western blotting. **(A)** Representative images of the blots. **(B)** Quantification of Iba1 immunoblots. Data were normalized by β-actin levels and expressed as fold-change relative to *Abi3*$^{+/+}$ (n=8/genotype). Data represent mean ± SEM. One-way ANOVA, Tukey's multiple comparison test; $p > 0.05$ between all comparisons.

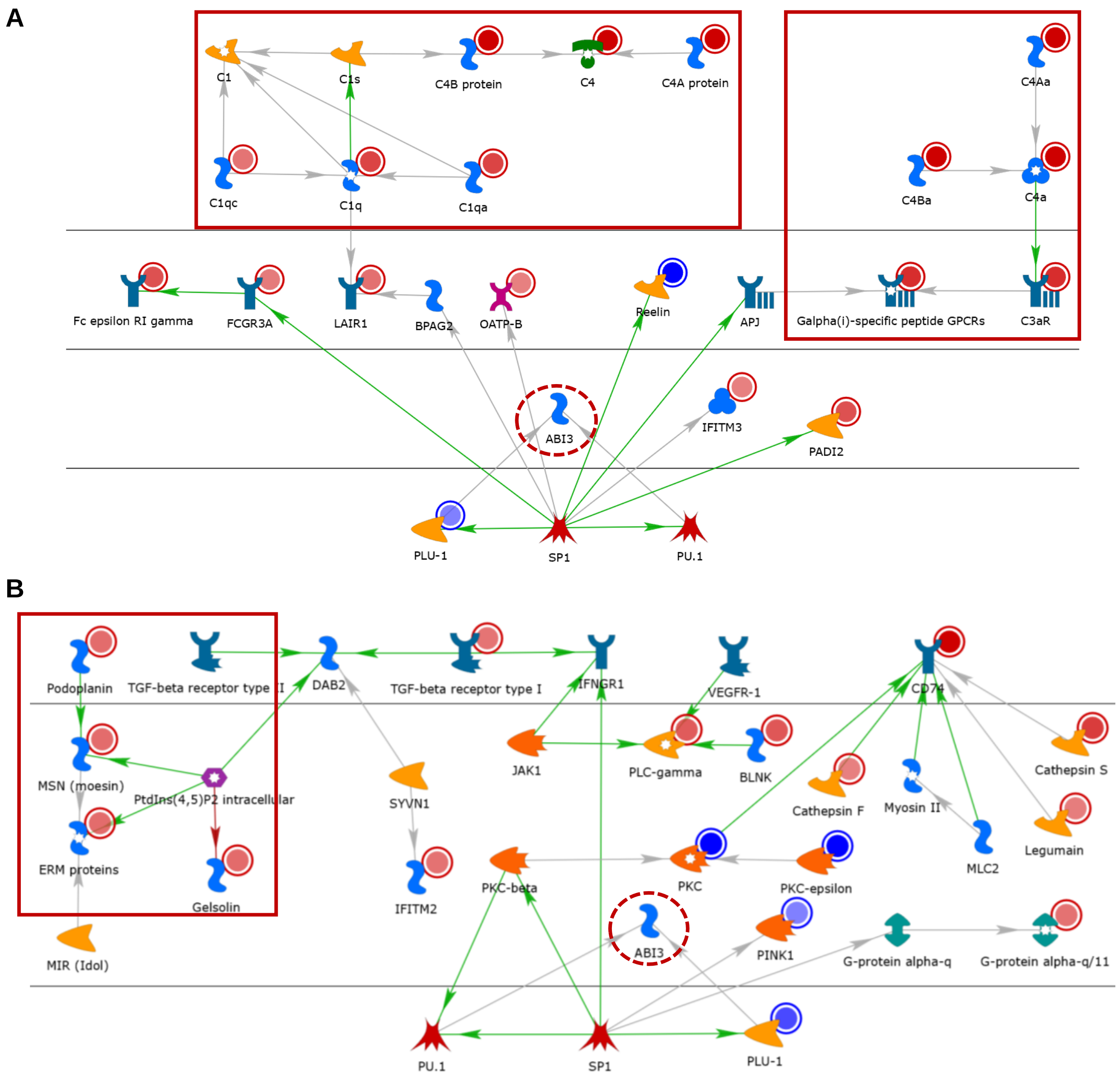

Fig. S7. Pathmap analysis of differentially expressed genes in *Abi3*$^{-/-}$ mice.

Differentially expressed genes (DEGs) were identified in the cortices of 8-month-old male *Abi3*$^{-/-}$ mice compared to *Abi3*$^{+/+}$ mice using nCounter NanoString mouse AD and Neuroinflammation panels (n=6/genotype). **(A-B)** Pathmap analyses were performed using MetaCore™. Upregulated genes in our dataset are shown with red circles and downregulated genes are shown with blue circles in the pathmaps. Green arrows between nodes represent activation, while grey arrows represent interaction with no specific direction of effect. DEGs shown within the red rectangles are involved in **(A)** complement cascade system and **(B)** actin cytoskeleton system.

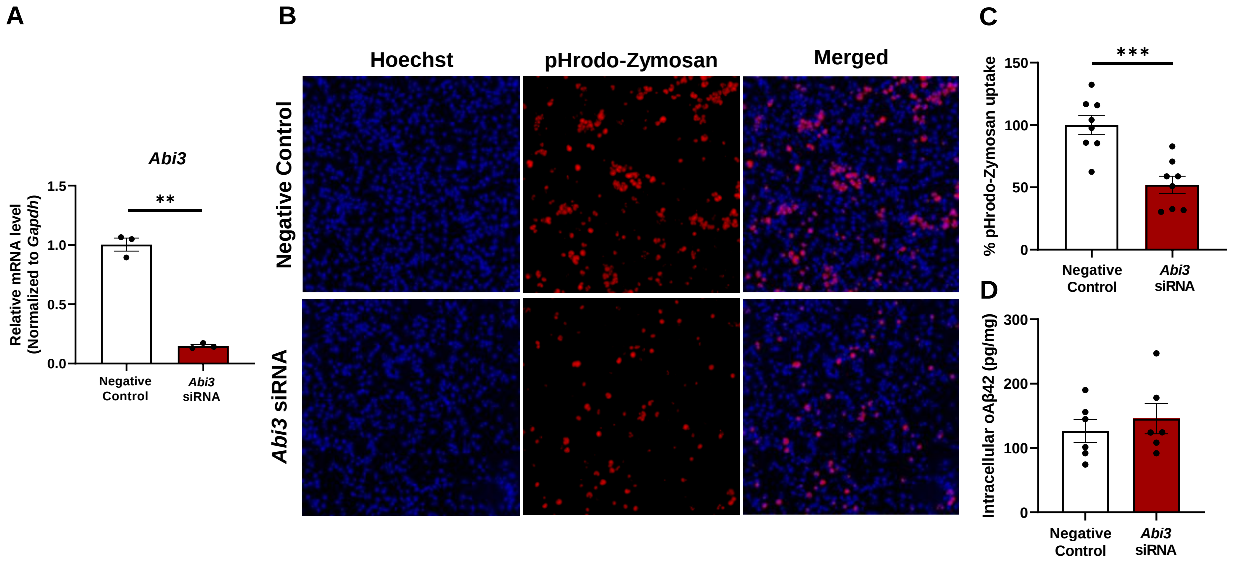

Fig. S8. Knock-down of *Abi3* decreases the phagocytosis of pH-rodo zymosan but does not affect Aβ uptake in microglia.

(A) BV-2 cells were transfected with *Abi3*-targeting or negative control siRNAs. *Abi3* siRNA treatment decreased mRNA level of *Abi3* by 85% (n=3/group). **(B)** 48h after transfection, cells were treated with pHrodo-labeled zymosan for 4h. Nuclei were stained with Hoechst. **(C)** The fluorescence intensity of pHrodo-zymosan was measured and normalized by nuclei staining. The percentage of pHrodo-zymosan uptake was decreased in *Abi3* siRNA-treated group compared to scrambled siRNA (n=8/group). Data represent mean ± SEM. Unpaired t-test; ***p<0.001. **(D)** Cells were treated with 200 nM oligomeric Aβ42 (oAβ42) and incubated for 1 h. After incubation, cells were collected to measure intracellular Aβ42 levels using the V-PLEX Plus Aβ Peptide Panel Kit. There was no difference between the groups (n=6/group). Data represent mean ± SEM. Unpaired t-test; p>0.05.

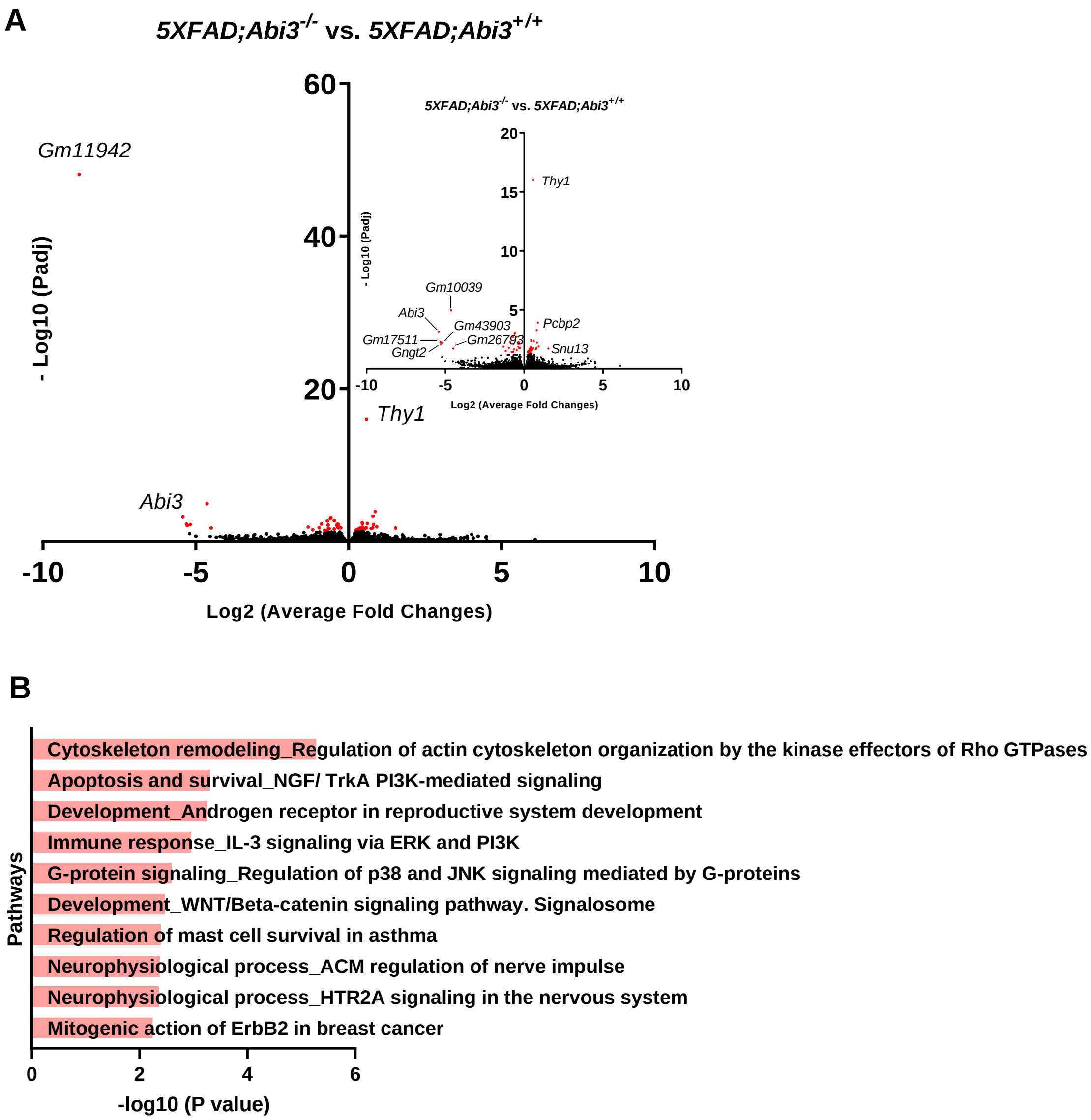

Fig. S9. Whole transcriptome analysis of *Abi3*$^{-/-}$ mice on 5XFAD background.

(A) Bulk RNA-sequencing was performed on the cortices of 8-month-old female *5XFAD*;*Abi3*$^{+/+}$ and *5XFAD*;*Abi3*$^{-/-}$ mice (n=6, *5XFAD*;*Abi3*$^{+/+}$; n=5, *5XFAD*;*Abi3*$^{-/-}$). Volcano plot shows the differentially expressed genes (DEGs). The *x*-axis shows the fold change and the y-axis shows the statistical significance level expressed as the $-\log_{10} P$ adjusted. The red dots represent genes significantly (p adj<0.05) up- or downregulated in *5XFAD*;*Abi3*$^{-/-}$ mice compared to *5XFAD*;*Abi3*$^{+/+}$ mice. (B) Pathway analysis was performed using the MetaCore™.
